# Fatty Acid Deaggregation and Extraction from Vesicles by Serum Albumin Proceeds Via a Dock-and-Load Mechanism

**DOI:** 10.1021/acs.jpclett.6c02325

**Published:** 2026-08-31

**Authors:** Sebastian Michler, Nico Schreiber, Taanya Kaur Saluja, Wai Sing Chan, Martin Gregor Knösche, Dariush Hinderberger

**Affiliations:** † 542351 Martin Luther University Halle-Wittenberg, Institute of Chemistry, Physical Chemistry−Complex Self-Organizing Systems, Von-Danckelmann-Platz 4, 06120 Halle (Saale), Germany

## Abstract

It is unknown whether the transport protein serum albumin extracts fatty acid (FA) monomers from aggregates and causes deaggregation. We studied the effect of human and bovine serum albumin on spin-labeled FAs 5/16-DOXYL stearic acid in phospholipid vesicles, in mixtures with decanoic acid (DA), and as pure aggregates. With continuous-wave electron paramagnetic resonance (CW EPR) spectroscopy, we distinguished and quantified free, aggregated, and albumin-bound FAs by their rotational dynamics, local concentrations, and polarities under different conditions. Our study unveils complex triangular equilibria between albumin and monomeric and aggregated FAs. The EPR spectra and altered aggregate sizes observed via dynamic light scattering confirm albumin–aggregate interactions. These argue for a dock-and-load FA transfer mechanism of albumin, while the binding of free FAs induces deaggregation. Similar effects on protein- or polymer-based aggregates could affect their use as drug carriers and guide future strategies in drug delivery or in the treatment of aggregation-related diseases.

## Graphical abstract

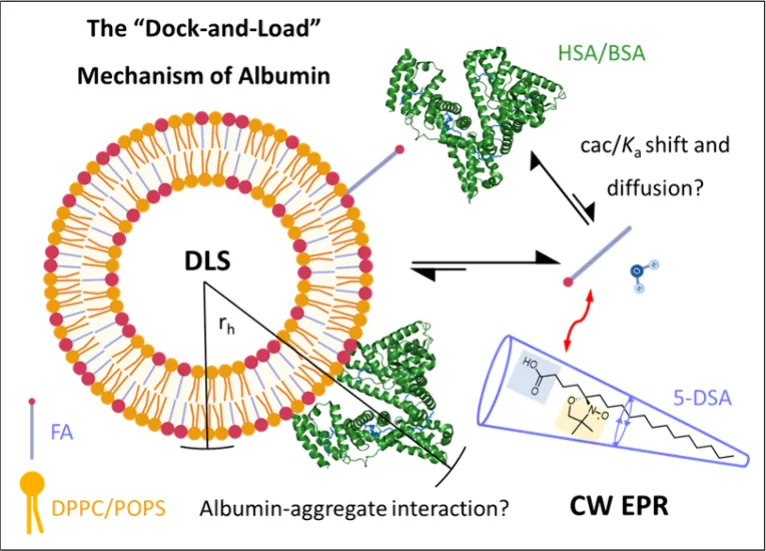

Serum albumin is a versatile transport protein that binds long- and medium-chain fatty acids (FAs) and has been utilized for applications like drug delivery, drug formulations or as a gel-based matrix.^1–6^ Despite its abundance and ample characterization in protein biophysics and biophysical chemistry,^7^ certain aspects of its functionalities remain unanswered so far. Does serum albumin actively interact with aggregates of FAs, phospholipids and other amphiphilic molecules? Is the protein able to extract monomeric FAs from these aggregates? We answer these questions in an EPR-spectroscopic study supported by DLS measurements. Conceivable interactions of serum albumin with FA aggregates and mixed FA-phospholipid vesicles in equilibrium with free FA monomers are illustrated in Figure 1. The (double) triangle equilibrium illustrates a simplified situation at the interface between cell membranes and blood plasma. Albumin occurs at high physiological concentrations of 530–750 μM^8^ in the human serum and binds long-chain FAs such as stearic acid (SA) with high binding affinities.^9^ Medium-chain FAs such as decanoic acid (DA) exhibit lower affinities but can be bound at even higher stoichiometries.^10^ Free FAs occur in an equilibrium with aggregates above their critical micellization (cmc) or aggregation (cac) concentration, while precipitation of crystalline FA occurs below their characteristic *Krafft* temperature due to their solubility limit. pH value and ionic strength of the solution also affect aggregation.^11,12^ While the monomer–aggregate equilibrium might be shifted due to albumin binding free FA, the proteins may also directly interact with the aggregates or extract FA monomers from them. The same interaction triangle appears for FAs incorporated into phospholipid liposomes. FAs have certain affinities to liposomes, depending on their chain length, headgroup and saturation. While FA binding of serum albumin was extensively studied in the past,^9,13–15^ aggregate^16–19^ and vesicle interactions^20–25^ of “XSA” (X = B or H) as well as mixed systems of FA and phospholipids with XSA^26–30^ were analyzed to a smaller extent and - to the best of our knowledge - never with EPR spectroscopy, except for pulsed EPR studies on HSA, but with different goals.^31^ Unlike many other techniques, EPR spectroscopy allows simultaneous quantification of free, vesicle- and albumin-bound FAs in equilibrium without separation protocols.

**Figure 1:**
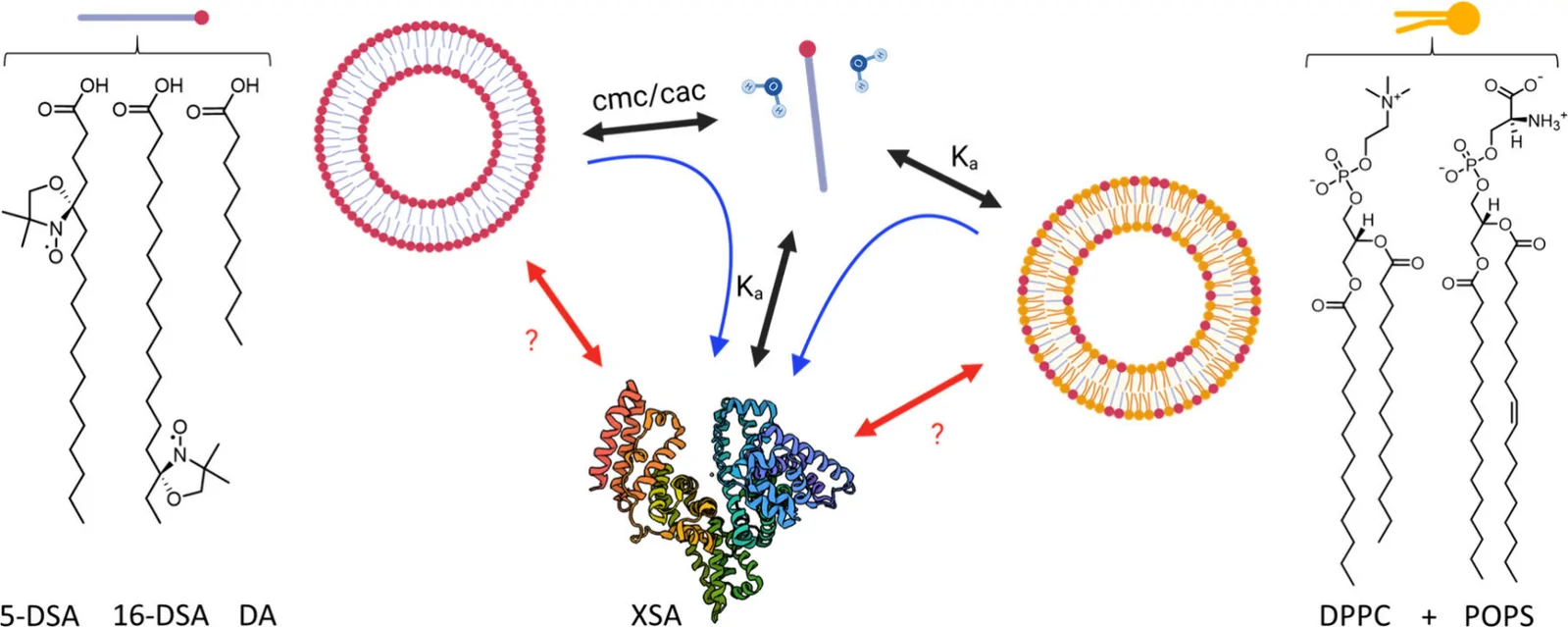
Triangle equilibrium between serum albumin (XSA, X = H (Human) or B (Bovine)), FA monomers (center) and FA aggregates (left) or liposome-bound FA (right). Red arrows indicate hypothetical albumin–aggregate interactions. Blue arrows illustrate the hypothetical extraction of FA monomers from aggregates by albumin. Black arrows indicate binding or aggregation equilibria. Chemical structures of the FAs and phospholipids used in this study are illustrated.

We analyze the spin-labeled (EPR-active) FAs 5- and 16-DOXYL stearic acid (5/16-DSA) incorporated in low concentrations into DPPC/POPS liposomes or DA aggregates. We observe changes of rotational dynamics and environmental polarities of the spin probes to analyze interactions within the mixed aggregates. Furthermore, we analyze the same probes dissolved alone in high concentrations above their cmc/cac, at which they assemble into micellar or vesicular structures.^32,33^ Local concentration enrichment in these FA aggregates leads to high interspin-collisional frequencies, detectable in CW EPR spectra through Heisenberg spin exchange broadening.^34,35^ XSA was added to all three systems to test for possible albumin–aggregate interactions in the triangle equilibria as well as a possible extraction of FA monomers from aggregates. The fundamental question of whether there are direct albumin-vesicle interactions may be pertinent to understand the transfer mechanism of FAs between albumin and cell membranes. Furthermore, native serum albumin might interfere with the stability of drug-loaded water–oil emulsions and polymeric micelles in the blood plasma.^17^

To analyze whether XSA extracts FAs out of aggregates, we first incorporated 5-DSA as model FA into phospholipid (DPPC/POPS 80/20 mol %) liposomes (small unilamellar vesicles, SUVs). Unlike FAs or lyso-phospholipids,^36^ phospholipid monomers are not expected to be extracted and bound by albumin, leaving these vesicles to remain stable with albumin. This study is especially relevant for a comparison with physiological systems, since phospholipid vesicles mimic the phospholipid part of cellular membranes. The transfer of FAs through membranes, for example from the extracellular serum to the intracellular cytoplasm, is supposed to occur mainly via flip-flop processes and to a lower degree actively with membrane transporters.^26^ In both cases, a transfer of the FA from the soluble carrier, such as albumin, or from the free state in the plasma to the lipid membrane is required. After crossing the membrane, intracellular carriers, such as FA binding proteins (FABPs), carry them to their intracellular operation sites. It was supposed that the FA transfer mechanism may be collision-mediated via direct surface interactions between complexed albumin and the outer membrane leaflet, or diffusion-mediated via free FA.^26^ It is reasonable to assume that the release of FA monomers from albumin at the membrane or their direct transfer requires an active or passive trigger, which might be controlled by concentration gradients of free FA, changes in local pH values^29^ or albumin-vesicle surface interactions.^26^ The ionic strength of the solution might affect electrostatic surface interactions and hydrophobic effects during collisional transfer.^26^ Binding of albumin to the membrane might catalyze dissociation of the albumin/FA complex, leading to a direct transfer of FAs to the vesicle without entering the pool of free FAs, since a diffusional mechanism alone would result in a lower FA transfer rate.^30,37^ Slightly higher local hydronium ion concentrations (“local pH”) might induce a release of albumin-bound FAs and diffusion-mediated transfer to vesicles.^39^ Despite these hypotheses there is a lack of experimental evidence for a coherent picture on the FA transfer.

In the CW EPR spectra of 5-DSA with liposomes we do not observe full incorporation of 5-DSA (Figure 2A,B), but 98.9% spin probes are liposome-bound (L) beside 1.1% free probes (F) at 37 °C, as quantified by spectral simulations (Figure S14, Table S4). This is expected due to the high partitioning preference of free FAs to enter liposomes.^28^ The doxyl group at carbon position 5 reports on the local environment and rotational mobility. Such, liposome-bound (L) and albumin-bound (A) FAs can be clearly distinguished due to the higher rotational dynamics (weaker interactions) but lower *a*_iso_ values (lower polarity) of (L) compared to (A) (Tables S2 and S3). After adding a low BSA concentration of 20 μM, an intermediate spectral species between (L) and (A) can be observed, showing a broadening compared to (L) while *A*_*zz*_ of (A) is not yet reached and the concentration of (F) is minimized compared to (A) and (L). Hence, free FAs seem to be bound with the highest affinity, but the appearance of this mixed species (AL) is clear evidence of direct albumin-liposome interactions (Figure 2C). The spectrum contains 47.2% (L) and 52.5% (AL). A simple binding of free FAs and shift of the (L)-(F) equilibrium would rather lead to a simple weighted superposition of (L) and (A) instead of a new species. Simulation residual plots with mixed and superimposed pure species in comparison validate the occurrence of the mixed species (Figure S15). For high XSA concentration (400 μM) in the range of physiological concentrations, the mixed spectra with liposomes look similar to the ones of 5-DSA with XSA alone, without much difference between BSA and HSA (Figure 2A,B). This clearly demonstrates a transfer of the FAs from liposomes to BSA. The mixed species (AL) at low BSA concentration indicates that this transfer occurs directly via protein–membrane interactions, but probably binding of free FAs and equilibrium shift also contribute. One may speculate that a full and immediate switch to albumin-bound FAs is unlikely achievable through shifts in the equilibrium between free and liposome-incorporated FAs or a concentration gradient alone. A selective extraction of FAs by albumin appears to be a more plausible explanation for this rapid and complete transfer, a potentially biologically relevant transfer pathway of FAs to/from XSA from/to the cellular membrane.

**Figure 2:**
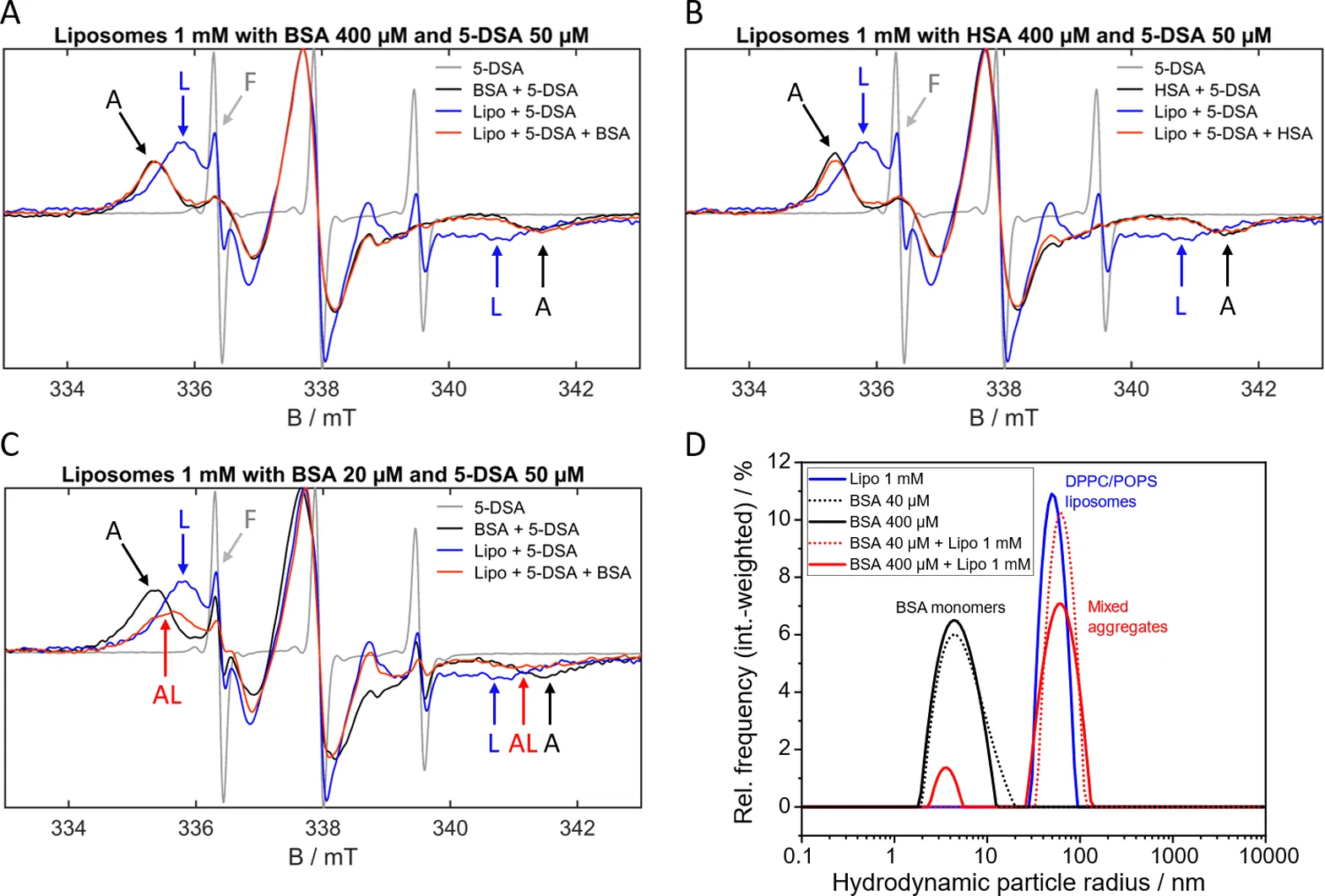
Liposomes with incorporated 5-DSA in mixtures with albumins. (A) CW EPR spectrum of 50 μM 5-DSA with 1 mM liposomes and 400 μM BSA. (B) Spectrum of 50 μM 5-DSA with 1 mM liposomes and 400 μM HSA. (C) Spectrum of 50 μM 5-DSA with 1 mM liposomes and 20 μM BSA. Due to normalization on the intensity maxima, EPR spectral intensities are not quantitatively comparable (normalization on the double integral: Figure S16). F = Free, A = albumin-bound, L = liposome-bound, AL = mixed component. (D) DLS particle radius distributions of BSA with 1 mM liposomes. All measurements were conducted at 37 °C. DLS distributions of liposomes with HSA are in Figure S1.

The particle radius distributions in Figure 2D show the peak of extruded DPPC/POPS liposomes, centered at 50 nm. Remarkably, the size peak becomes broader and the maximum is ∼ 10 nm higher after addition of BSA (and HSA as well). Filtrated BSA (100 nm cutoff) exhibits only a broad peak of protein monomers with a maximum at 3.7 nm, but no larger protein aggregates occur, while HSA reassembles a small fraction of aggregates even after filtration (see SI) which disappear in a mixture with liposomes. XSA monomers are still visible after mixing with liposomes, but to a much smaller extent. These observations again suggest a certain fraction of XSA molecules interacting with the DPPC/POPS liposomes, possibly assembling at the liposome surfaces. Interactions of HSA/BSA with different, even negatively charged,^23^ phospholipid vesicles were shown by various other methods, and explained by electrostatic and hydrophobic effect-driven interactions of amino acids with the liposome surface.^20–25^ As an additional check, we prepared liposomes containing 100 μM 5-DOXYL-PC and added 400 μM XSA. The spin-labeled lipids are fully incorporated into the liposomes (Figure S2). Especially at 37 °C the spectra from the mixtures with albumins deviate slightly from the pure liposomes (very similar for both, HSA and BSA). This confirms the existence of FA-independent albumin-liposome interactions. But since the spectra of 5-DOXYL-PC in liposomes are already strongly broadened due to lipid immobilization, changes after XSA addition are not well resolved and we refrain from conclusions about the type and strength of these interactions. CD spectroscopy demonstrates that the α-helix content decreases by 27.1% and 21.2% at 37 °C for BSA and HSA, respectively, in the presence of liposomes, indicating structural changes of albumins during their interaction with liposomes (Figures S18 and S19, Table S7).

After studying the effect of albumins on phospholipid vesicles we extend this approach to mixed aggregates of medium chain-length decanoic acid (DA) and 5 or 16-DSA, respectively. In this series of experiments, low concentrations of spin probes were incorporated into highly concentrated DA aggregates. DA is bound by albumin with high binding stoichiometry but moderate affinity, adding a potential competition in binding for the DSA probe molecules. A crystal structure of the DA-HSA complex has been solved before and reveals at least ten bound DA molecules^10^ (Figure 3E) with a *K*_d_ of 740 μM.^40^ The affinity is much lower than for HSA/SA (*K*_d_ = 7–360 nM^9^) or BSA/SA (*K*_d_ = 14 nM^41^), although only 7 SA molecules are bound per protein. With a *K*_d_ of 185 nM for HSA at 37 °C and 603 nM at 25 °C,^42^ the 16-DSA affinity is lower than for native SA but still much higher than for DA. For 5-DSA/HSA binding, *K*_d_ values of 0.2–158 μM depending on the binding site are reported.^33^ The CW EPR spectra of mixed DA/5-DSA systems (Figure 3A–D) reveal no free 5-DSA but a strongly anisotropic component (D), originating from 5-DSA, incorporated into DA aggregates, which assembled, since the DA concentration of 50 mM was chosen to be above its cac of 29 mM.^43^ The cac and the nature of the aggregates are pH-dependent. While high pH values promote the formation of micelles due to the deprotonation of the monomers and their electrostatic repulsion, a pH closer to the p*K*_A_ value of DA (6.8 for aggregated,^12^ 4.9 for monomeric DA^44^) enables the formation of multilayered vesicles due to stabilizing hydrogen bonds between protonated and deprotonated monomers.^11^

**Figure 3:**
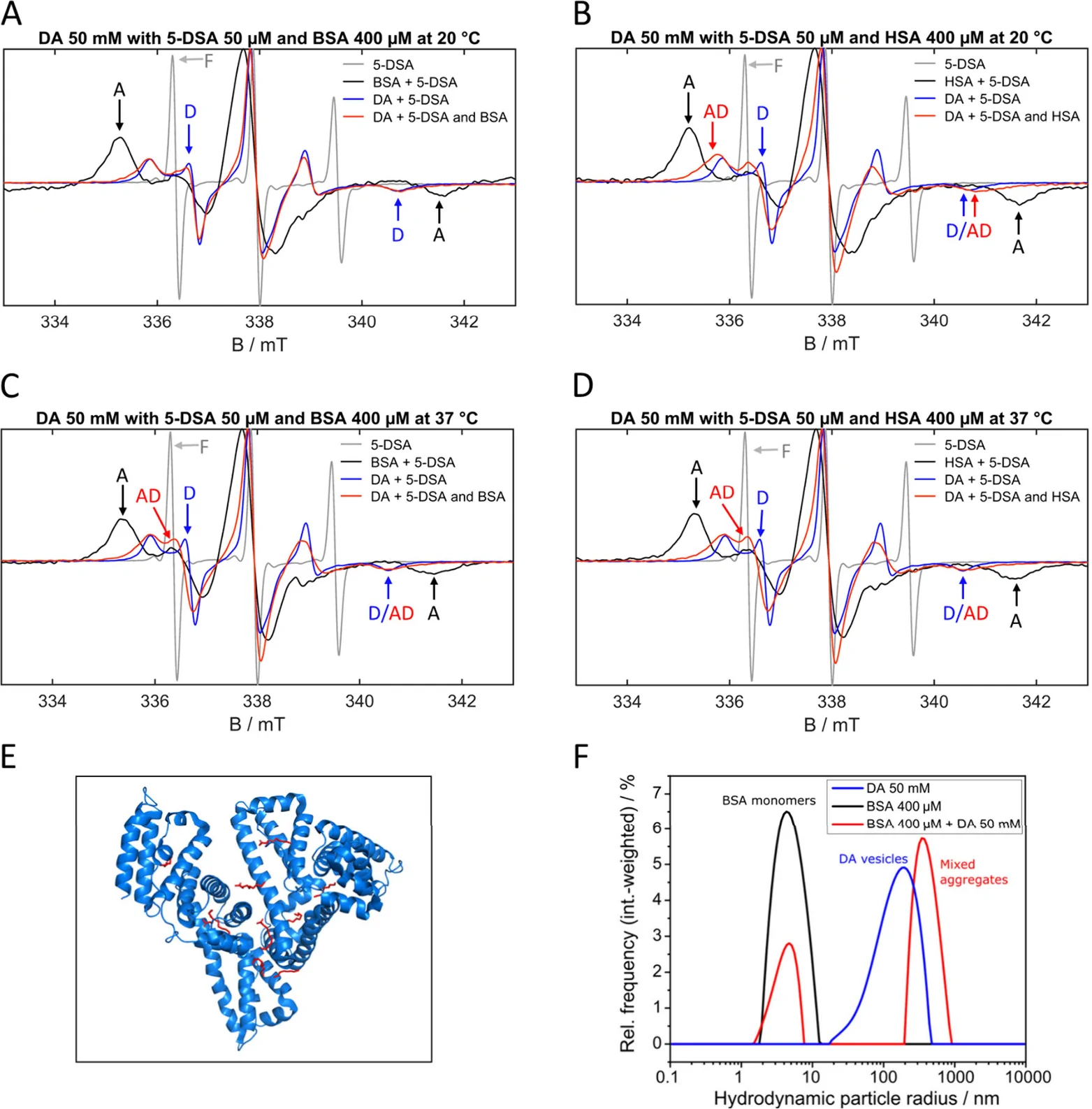
CW EPR spectra of DA with 5-DSA and XSA. DA vesicles with 5-DSA and BSA (A) or HSA (B) at 20 °C, as well as at 37 °C (C, D). (E) Crystal structure of DA-complexed HSA.^10^ (F) DLS particle radii of BSA, DA, and their mixture. Distributions with HSA can be found in Figure S3; CW EPR spectra of 16-DSA with DA and BSA can be found in Figure S5. Due to normalization on the intensity maxima, EPR spectral intensities are not quantitatively comparable here (normalization on double integrals: Figure S17). F = Free, A = albumin-bound, D = DA-bound, AD = mixed component.

The addition of 400 μM XSA changes the shape of the spectrum but it remains different from the one of pure albumin-bound 5-DSA (A). It is not a sole superposition of (A) and (D) (see Figure S15), indicating an interaction of albumins with the DA vesicles, leading to a new spectral species (AD) which is slightly more polar than (D) but broader (Table S2). Interestingly, with HSA a small proportion of free 5-DSA appears (F) in the spectra, possibly due to partial deaggregation of DA, leading to the release of 5-DSA. (F) may not be bound by HSA due to the excessive free DA, saturating the binding sites of HSA. (AD) is especially present at 37 °C for both XSA, while it seems to be rather close to the (D) species at 20 °C for BSA, but not for HSA. Possibly, albumins have the highest activity in aggregate interactions at physiological temperature, while HSA shows a stronger effect also at lower temperature, maybe due to a higher structural flexibility and more hydrophobic surface clusters.^45^ CD spectra reveal slight reductions of the α-helix percentage of XSA in the presence of DA (Figures S18–19, Table S7). The particle radius distributions from DLS measurements (Figure 3F) show a broad peak for pure DA aggregates, ranging from 20 to 500 nm with a small (reproducibly measurable) shoulder at smaller radii and an intensity maximum at 195 nm. BSA solutions that were filtrated through a 100 nm pore syringe filter prior to the DLS measurement show only protein monomers or a mixture of monomers and dimers with an intensity maximum at 4.4 nm. In the mixture with the DA aggregates this peak remains at a similar particle radius but becomes much smaller in its weighting. On the other hand, the vesicle peak becomes sharper and the peak maximum is shifted to a larger radius of 350 nm, the previous shoulder at lower radii disappears.

Small deviations in the spectra occur depending on the mixing sequence of the components (Figure S4). While premixing of 5-DSA with DA or BSA results in the same spectrum, premixing of DA and BSA leads to a broader spectrum at 20 °C, possibly due to a combination of 5-DSA immobilization in vesicles and in BSA. 16-DSA with DA aggregates and BSA exhibits the same influence of the mixing sequence (Figure S5) but the spectra show less pronounced immobilization. CW EPR temperature series reveal complex temperature-dependent dynamics and exchange between (F), (A) and (D) (Figure S6). From −30 to 0 °C, 5-DSA remains immobilized but the residual dynamics increase. The spectral shape and *A*_*zz*_ value compared to pure 5-DSA in frozen solution suggests predominantly albumin-bound ligands (A) at low temperatures. Free 5-DSA (F) appears in low amounts at 0 and 5 °C but disappears again at 10 °C, the expected *Krafft* temperature of DA.^46,47^ DA aggregates (D) form and all free and BSA-bound 5-DSA, (F) and (A), vanish completely. FA have a higher thermodynamic preference to incorporate into the newly formed DA aggregates than to stay in the binding pockets of albumin, which is likely since the aggregate environment is more hydrophobic than the binding pockets, as confirmed by the smaller *A*_*zz*_ value of (D). Here, the binding processes of XSA with 5-DSA and with DA, respectively, are in competition. We do not observe complete extraction of 5-DSA from DA aggregates in this concentration regime, as compared to the liposomes.

Finally, we study the aggregation of pure 5- and 16-DSA in dependence of concentration, pH-value and temperature via CW EPR spectroscopy. Their aggregation can be detected through the occurrence of a strongly broadened spectral component co-appearing with the sharp, nearly isotropic signals of the free probes (Figure 4). This broadening originates from the Heisenberg spin exchange effect of overlapping singly occupied molecular orbitals (SOMO).^35^ The exchange frequency ν_ex_ depends on the intermolecular collisional frequency, which itself is a measure of the local concentration of spin probes in the aggregates. A higher total and local spin probe concentration leads to a higher exchange frequency and a larger fraction of the aggregated spectral component compared to the free probes (Figure S7). The Heisenberg spin exchange crucially depends on pH and temperature,^48–51^ e.g., the collisional frequency increases with increasing temperature (Figure S9). Since the charges are pH-dependent, there is a nontrivial pH dependence of spin exchange. By increasing pH, the increasing number of charged carboxylic acids does not only lead to increased charge repulsion but also to clearer formation of micelles or vesicles of 5/16-DSA (Figure S11). Spectral simulations indicate, that residual dipolar couplings are negligible in the high concentration samples since the residence times are too short and dipolar broadening effectively needs a near rigid limit state (Figures S20 and S21, Table S8).

**Figure 4:**
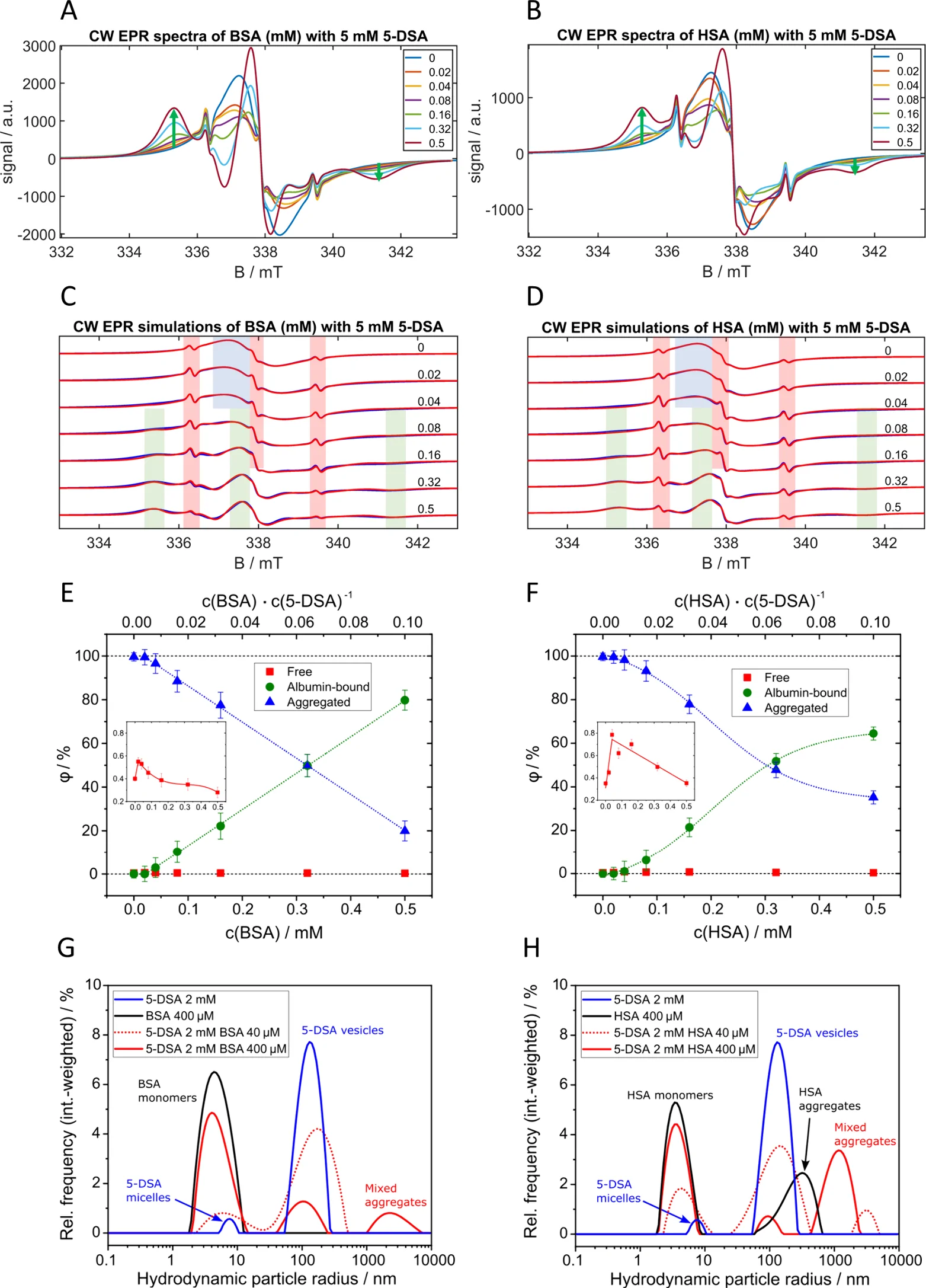
CW EPR spectra of 5-DSA (5 mM) with different concentrations of BSA and HSA at 37 °C and pH 7.4. (A, B) CW EPR spectra with measured intensities, green arrows indicate increasing albumin-bound component. (C, D) Stacked CW EPR spectra (blue) with spectral simulations (red). Red bands = free, blue = aggregated, green = albumin-bound 5-DSA. (E, F) Component proportions as functions of XSA concentrations. Zoomed insets in (E) and (F) highlight the development of free 5-DSA. The RMSD values from the simulation served as error bars for albumin-bound and aggregated 5-DSA, while RMSD/100 was used for free species due to their higher sensitivities and different proportion magnitudes. (G, H) DLS particle radius distributions. CW EPR spectra and radius distributions with 16-DSA, and distributions with 40 μM XSA can be found in Figures S12 and S8.

To analyze the effect of XSA interacting with the aggregated spin probes, we mixed 5- and 16-DSA, respectively, at 5 mM, which is above their cac (35 μM for 5-DSA,^33^ 285 μM for 16-DSA^42^), with the proteins. Hence, the FA probes form large aggregates to a sizable degree with well-observable exchange-broadened EPR spectra. Since among the two probes 5-DSA exhibits a higher percentage of aggregated probes, we will focus on this probe interacting with both albumins (Figure 4), while similar series for 16-DSA and other conditions can be found in the SI. Spectral simulations show that 16-DSA has a higher exchange frequency than 5-DSA at 5 mM (Table S2), being in the exchange narrowing regime, probably due to smaller distances and higher local concentrations and mobilities of doxyl groups in 16-DSA aggregates (Figure S13). The concentration-dependent CW-EPR spectra and spectral simulations in Figure 4C,D indicate a transition from exchange-dominated spectra, stemming from FA aggregates (E) at low albumin concentration, to spectra, indicating rotationally immobilized, albumin-bound FAs (A) at high albumin concentrations. The small fraction of free FAs (F) goes through a maximum in the middle of the concentration series. Similar processes can also be observed for other pH values and for XSA concentration series with 16-DSA (Figures S11, S12). The exchange frequency of (E) decreases with increasing XSA content, demonstrating reducing amounts of aggregated radicals through XSA insertion or FA extraction. HSA and BSA exhibit a similar general mechanism but the percentage of (A) increases linearly for BSA, while it follows a sigmoidal increase for HSA (Figure 4E,F). A larger proportion of (E) remains at high albumin content in case of HSA and hence the deaggregation is less effective. Yet, the 50% equilibrium state of (A) and (E) is reached at the same XSA concentration of 0.32 mM (= 15.6-fold FA excess). The decrease of ν_ex_ (Tables S5, S6) and the low transient concentration of (F) do not argue for a simple cac-equilibrium shift and diffusion of free FAs but rather for direct interactions of XSA with aggregates promoting an extraction of FAs.

So far, we used the expression “aggregates” to describe a wide variety of possible molecular assemblies. However, the nature of 5- and 16-DSA aggregates can be more precisely described as smaller micelles or larger uni- or multilamellar vesicles. The particle radii of the aggregates measured by DLS (Figure 4G,H) rather indicate the formation of larger vesicles since hydrated micelles with a single spherical layer of 5/16-DSA would have a lower radius of approximately 2–10 nm.^52^ The DLS results exhibit different radius distributions and weightings for 5- and 16-DSA, indicating that the doxyl group position affects aggregate geometry. In case of 5-DSA, a small fraction of micelles (8 nm) seems to appear besides larger vesicles (100 nm), while 16-DSA shows two different vesicle species (100 and 600 nm) with broad size distributions, but no micelles. Unlike in the rather conically shaped 5-DSA, the bulky doxyl group at the chain end of 16-DSA prevents micelle formation (Figure S13). The difference in light scattering is even visible without DLS, since a 2 mM 5-DSA solution with pH 7.4 is clear, while a 16-DSA solution is turbid under identical conditions. The pH value, relative to the p*K*_A_ value of the FA solution, affects stability, size and lamellarity of FA aggregates.^11,12^ Since 5/16-DSA at pH 7.4 are relatively close to their p*K*_A_ value (6.55 for 5-DSA^53^), a significant fraction may form hydrogen-bonded dimers as discussed above for DA. Due to 16-DSA’s rather rectangular than conical geometry, these dimers prefer assembling into larger vesicles in solution, similar to phospholipids.

A turbid 5 mM 16-DSA solution becomes clear after addition of at least 700 μM BSA (Figure S10), proving solubilization or at least restructuring of the aggregates by BSA. This observation was confirmed by light transmittance measurements during DLS experiments (Table S1) and is reflected in particle radius distributions. The addition of 40 μM XSA leads to a broadening of the 5-DSA vesicle peak at around 100 nm, arising from the intermediate formation of medium-sized mixed aggregates. But with 400 μM of XSA, mostly protein monomers and larger mixed aggregates remain while the original medium-sized vesicles almost disappear. This mechanism is found for both albumins. HSA protein aggregates seem to disappear as well after mixing with 5-DSA. The aggregate-albumin interaction could also have a deaggregating effect on the protein itself. In case of 16-DSA, the large aggregates disappear but mixed aggregates with a medium radius become dominant (Figure S8 **)**. With 400 μM BSA almost only protein monomers remain, again indicating not only protein-aggregate interactions but also an efficient deaggregation effect. Combined CW EPR and DLS results deliver strong evidence that also aggregates consisting of pure FA species interact with BSA and HSA. This interaction may effectively trigger active extraction and binding of FA, finally leading to a deaggregation and solubilization of aggregates.

This study explores the complex triangle equilibria between FA monomers, aggregates/liposomes and albumin. We found clear evidence by EPR and DLS experiments that albumin has a deaggregating effect and direct albumin–aggregate interactions occur within different systems, leading to FA extraction. This goes beyond the expected monomer–aggregate equilibrium shift through the binding of free FA monomers. We observed the formation of mixed aggregates, as the hallmark of a working principle that we summarize as a (collisional) dock-and-load-mechanism. This effect was found for pure and mixed FA aggregates consisting of monomers that are bound by albumins themselves, and also for liposomes which incorporated FAs but consisted of lipids that are not bound by XSA. HSA and BSA show a similar general mechanism but slight differences in the extraction efficiency. The equilibrium and interaction patterns found in the three systems analyzed are illustrated in Figure 5.

**Figure 5:**
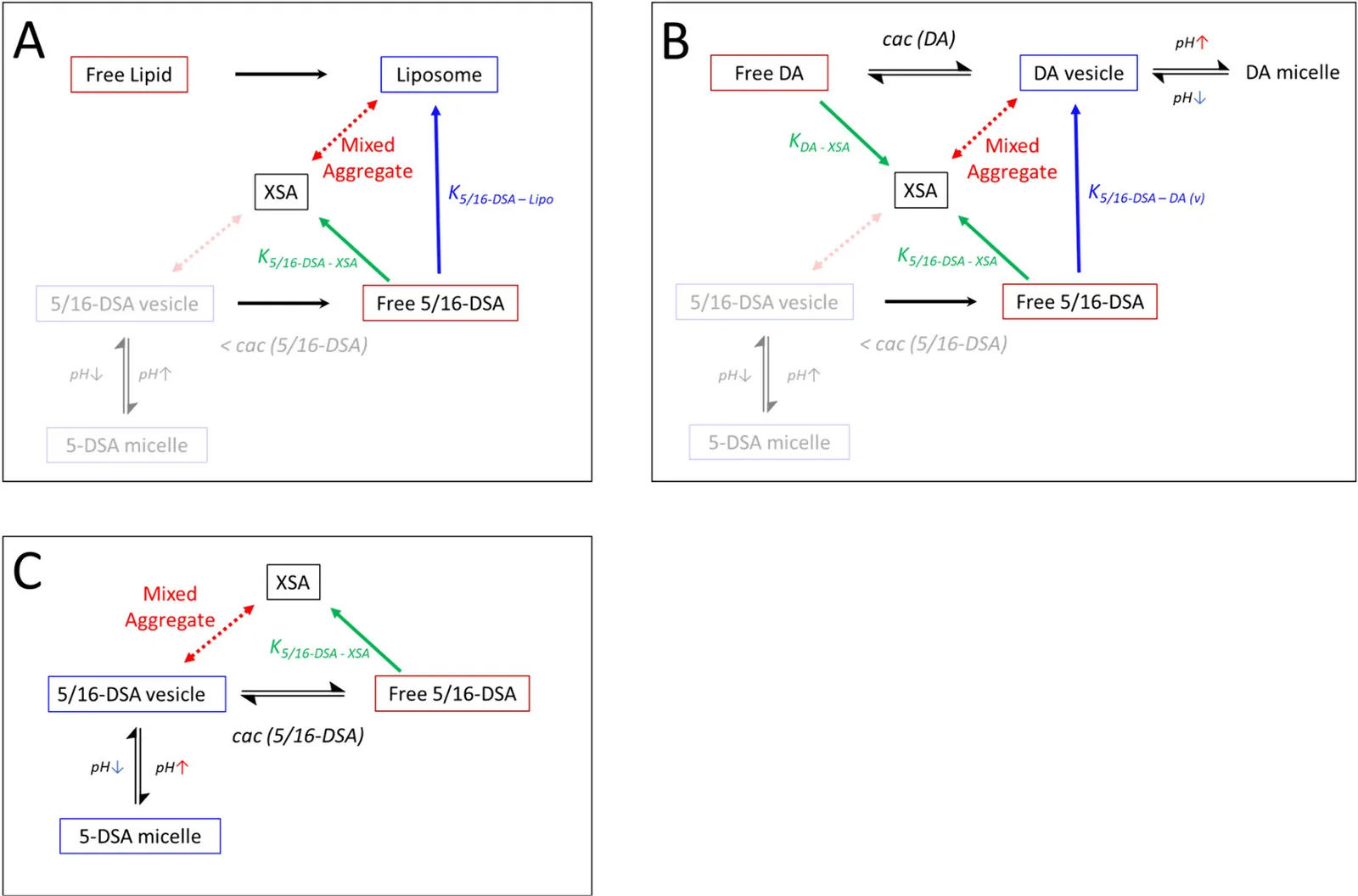
Equilibrium schemes of all analyzed systems. (A) Low-concentration 5/16-DSA with XSA and phospholipid vesicles. (B) Low-concentration 5/16-DSA with XSA and DA. (C) High-concentration 5/16-DSA with XSA.

Our findings shed light on the FA transfer mechanism from plasma to cells and the applications of aggregate-based drug carriers in the blood. The deaggregating effect of albumin might represent an additional intrinsic function of this protein class. The thermodynamics of the equilibria could be analyzed with ITC measurements, although the high system complexity would make their analysis challenging, or with our recently developed EPR-STAMP methodology.^54^ The dynamics of micelles and vesicles prepared from DA require a more profound EPR spectroscopic analysis to understand the complex concentration-, pH- and temperature-dependent equilibria of this system. This is particularly of high interest since DA aggregates are a common model for prebiotic protocells.^11^

## Experimental Section

Detailed descriptions of the experimental procedures, methods, and materials can be found in the SI.

## Supplementary Material



## Notes

Description of the Materials and Experimental Procedures; Figure S1: DLS particle radius distributions of DPPC/POPS liposomes HSA; Figure S2: CW EPR spectra of DPPC/POPS liposomes with 5-DOXYL-PC; Figure S3: DLS particle radius distributions of DA with HSA; Figure S4: CW EPR spectra of DA aggregates mixed with 5-DSA and XSA; Figure S5: CW EPR spectra of DA aggregates mixed with 16-DSA and BSA; Figure S6: Temperature dynamics of mixed DA/5-DSA aggregates in interaction with BSA; Comment on the temperature dependence of DA/XSA mixtures; Figure S7: Concentration-dependent aggregate formation of 5-DSA; Figure S8: DLS particle radius distributions of 16-DSA vesicles with XSA; Figure S9: Temperature dynamics in the CW EPR spectra of different aggregated systems; Comment on the temperature dynamics of 5/16-DSA aggregates; Figure S10: Turbidity comparison of different vesicle/BSA-containing solutions; Table S1: Detected light transmittance during DLS measurements for various samples; Figure S11: pH-dependent CW EPR concentration series of BSA with 5 mM 5-DSA; Figure S12: CW EPR spectra of concentration series of XSA with 5-DSA and 16-DSA; Figure S13: Simplified schemes of micellar or vesicular structures of 5- and 16-DSA; Figure S14: CW EPR spectra and simulations of different systems; Figure S15: Comparison of CW EPR simulations with pure and mixed components; Figure S16: DI-normalized liposome-related CW EPR spectra from Figure 2; Figure S17: DI-normalized DA-related CW EPR spectra from Figure 3; Comment on the simulations of mixed components; Table S2: Simulated parameters of different spectral components; Table S3: Apparent isotropic hyperfine coupling constants and rotational correlation times; Table S4: Component proportions of selected samples with mixed spectral species; Table S5: Simulated parameters of the BSA concentration series; Table S6: Simulated parameters of the HSA concentration series; Figure S18: CD spectra of various systems at 25 °C; Figure S19: CD spectra of various systems at 37 °C; Table S7: Estimated percentages of secondary structural elements from CD spectra; Comment on the CD spectra; Figure S20-21: Simulations of 5-DSA with exchange and dipolar coupling; Table S8: Results of the simulations with dipolar coupling; Comment on the simulations of dipolar coupling; Supporting References (PDF)
