# Supplementary material for: Fatty Acid Deaggregation and Extraction from Vesicles by Serum Albumin Proceeds Via a Dock-and-Load Mechanism
Source: J Phys Chem Lett. 2026 Aug 31;17(36):10509–17. doi: 10.1021/acs.jpclett.6c02325 (PMC13573608; doi:10.1021/acs.jpclett.6c02325)
Supplement: Supplementary file 1 [file jz6c02325_si_001.pdf]

Supporting Information

For the manuscript:

Fatty Acid Deaggregation and Extraction from Vesicles by Serum Albumin Proceeds Via a Dock-and-Load Mechanism

Sebastian Michler,[1] Nico Schreiber,[1] Taanya Kaur Saluja,[1] Wai Sing Chan,[1] Martin Gregor Knösche,[1] Dariush Hinderberger*,[1]

[1]Martin Luther University Halle-Wittenberg, Institute of Chemistry, Physical Chemistry – Complex Self-Organizing Systems, Von-Danckelmann-Platz 4, 06120, Halle (Saale), Germany

**to whom correspondence should be addressed: dariush.hinderberger@chemie.uni-halle.de*

Contents of the Supporting Information

Description of the Materials and Experimental Procedures

Figure S 1: DLS particle radius distributions of DPPC/POPS liposomes HSA.

Figure S 2: CW EPR spectra of DPPC/POPS liposomes with 5-DOXYL-PC.

Figure S 3: DLS particle radius distributions of DA with HSA.

Figure S 4: CW EPR spectra of DA aggregates mixed with 5-DSA and XSA.

Figure S 5: CW EPR spectra of DA aggregates mixed with 16-DSA and BSA.

Figure S 6: Temperature dynamics of mixed DA/5-DSA aggregates in interaction with BSA.

Comment on the temperature dependence of DA/XSA mixtures.

Figure S 7: Concentration-dependent aggregate formation of 5-DSA.

Figure S 8: DLS particle radius distributions of 16-DSA vesicles with XSA.

Figure S 9: Temperature dynamics in the CW EPR spectra of different aggregated systems.

Comment on the temperature dynamics of 5/16-DSA aggregates.

Figure S 10: Turbidity comparison of different vesicle/BSA-containing solutions.

Table S 1: Detected light transmittance during DLS measurements for various samples.

Figure S 11: pH-dependent CW EPR concentration series of BSA with 5 mM 5-DSA.

Figure S 12: CW EPR spectra of concentration series of XSA with 5-DSA and 16-DSA.

Figure S 13: Simplified schemes of micellar or vesicular structures of 5- and 16-DSA.

Figure S 14: CW EPR spectra and simulations of different systems.

Figure S 15: Comparison of CW EPR simulations with pure and mixed components.

Figure S 16: DI-normalized liposome-related CW EPR spectra from Figure 2.

Figure S 17: DI-normalized DA-related CW EPR spectra from Figure 3.

Comment on the simulations of mixed components.

Table S 2: Simulated parameters of different spectral components.

Table S 3: Apparent isotropic hyperfine coupling constants and rotational correlation times.

Table S 4: Component proportions of selected samples with mixed spectral species.

Table S 5: Simulated parameters of the BSA concentration series.

Table S 6: Simulated parameters of the HSA concentration series.

Figure S 18: CD spectra of various systems at 25 °C.

Figure S 19: CD spectra of various systems at 37 °C.

Table S 7: Estimated percentages of secondary structural elements from CD spectra.

Comment on the CD spectra.

Figures S 20-21: Simulations of 5-DSA with exchange and dipolar coupling.

Table S 8: Results of the simulations with dipolar coupling.

Comment on the simulations of dipolar coupling.

Supporting References

Description of the Materials and Experimental Procedures

Sample preparation

Stock solutions of essentially fatty acid free human and bovine serum albumin (HSA/BSA, Sigma Aldrich, purified from serum) with concentrations of 1 mM were prepared in 0.137 M DPBS buffer with pH 7.4 or in ultrapure water and filtrated through a 100-nm cut-off syringe filter. The concentrations were checked with a DS-11 microvolume spectral photometer (DeNovix Inc.) by measuring the light absorbance at 280 nm. All samples were prepared in low-binding PCR tubes by mixing the stock solutions of BSA/HSA (final concentration: 400, 40 or 20 µM, except for concentration series) and 5- or 16-DSA (final concentration: 2 or 5 mM), setting the pH value and filling up with buffer to obtain the desired final concentration.

5- and 16-DOXYL stearic acid (5/16-DSA, Sigma Aldrich/ Avanti Polar Lipids) were dissolved in 0.1 M KOH solution with soft ultrasonification to obtain 26 mM stock solutions. While 16-DSA was purchased as free fatty acid, 5-DSA was used as an ammonium salt.

Decanoic acid (DA, Sigma Aldrich) was dissolved in 0.19 M NaOH solution at 40 °C with 30 min of ultrasonic treatment to obtain a 180 mM stock solution which was filtrated through a 450-nm cut-off syringe filter. DA-containing samples were prepared in water to exclude an additional aggregating effect of Ca^{2+} or Mg^{2+} ions which are present in the DPBS buffer. But a Na^{+} concentration of 190 mM was set consistently with 1 M NaCl solution since the ionic strength can change due to the pH titration and could affect the aggregate formation.

DPPC/POPS liposome solutions (80/20 mol-%, 4 mM or 3 mM stock solutions) were prepared by mixing stock solutions of DPPC and POPS (both from Avanti Polar Lipids Inc.) in certain ratios, co-dissolving 400 µM of 5-DOXYL-PC (16:0, Avanti Polar Lipids Inc.) or 5-DSA for certain samples, evaporating the $CHCl_3$/MeOH under a gentle stream of argon and resuspending the films in DPBS buffer or water. This resulted in 1 mM or 1.5 mM liposome and 50-100 µM 5-DSA or 5-DOXYL-PC final concentrations in the samples. Alternatively, 5-DSA was added to the final liposome solution in buffer, resulting in less effective incorporation of 5-DSA as checked via EPR measurements. Hence, we co-dissolved 5-DSA directly with the lipids instead. The liposome solutions were extruded through 100-nm diameter PCTE membranes (GVS Filter Technology) at 45 °C and the particle size distribution was checked via DLS. The pH values in the final samples were set by titrating small volumes of acidic and basic DPBS buffer or NaOH/HCl and filling up with water or pH 7.4 buffer.

Stock solutions were mixed and pH values set to obtain the final samples. All substances were used without further purification.

CW-EPR spectroscopy and simulations

EPR-silent quartz glass capillaries (*BLAUBRAND®* IntraMARK) were loaded with solution and sealed with *Critoseal®*. CW EPR measurements were conducted at a benchtop spectrometer MS5000 (Bruker Biospin, formerly Freiberg Instruments) which operates at X-band frequency. Concentration-dependent series were measured at 20 °C and 37 °C. Temperature sequences were measured from -30 °C to 90 °C with measurements every 5 or 10 °C. All measurements consisted of 10 scans with 60 s of measuring time, applying a field sweep from 332 to 344 mT. A modulation amplitude of 0.1 mT, a modulation frequency of 100 kHz, a microwave power of 4.79 mW and a digital RC filter with a filter parameter of 0.2 was used. The CW EPR spectra were summed up, analyzed and simulated in Matlab R2026a with the Easyspin package (easyspin-6.0.0-dev.43)[1] and the Easyspin function *Chili* for spectra in the slow-motional regime as developed by Schneider and Freed[2, 3]. For a better visualization, some spectra were normalized on the maximum intensities or on their double integrals (**Figures S 16-17**). Root mean square deviations (RMSD) were extracted as error values from the simulations. Simulations with contributing dipolar coupling were performed as described in the comment below **Table S 8**.

Dynamic light scattering (DLS)

DLS measurements were conducted with a Lightsizer 500 device (Anton Paar, Graz, Austria) with the software *Anton Paar Kalliope*. 60 µL of sample solutions were filled into quartz low-volume cuvettes. The measurements were done at 37 °C or 20 °C with an equilibration time of 1 min, a measurement time of 30 s and 6 runs, using side- (90°) and back- (175°) scattered light with a wavelength of 658 nm. Side- and back-scattered light delivered similar radius distributions for most systems. Volume-weighting resulted in similar radii and comparable general trends but for polymodal distributions lower radii were visualized unrealistically over-weighted in the resulting Figures. For further analysis, only the intensity-weighted distributions with side-scattered light were used.

Circular dichroism (CD) spectroscopy

Far-UV CD spectroscopy was measured with a Jasco J-815 CD spectrometer, using light of a wavelength from 190 to 250 nm, a scanning speed of 50 nm/min and averaging over three scans. A 0.1 mm quartz glass cuvette (Hellma Analytics) was used. The cuvette was filled with 40 µL of sample solution, avoiding air bubbles, and it was cleaned after each measurement with ethanol and water. 0.05 M DPBS buffer or water (for DA-containing samples) was used as the sample medium. XSA concentrations were set to 10 µM and spectra were measured at 25 and 37 °C. Measurements were also performed with 100 µM XSA concentration but the high protein concentration resulted in distortions of the α-helix peaks. All samples were set to pH 7.4. Background spectra of pure DPBS buffer and water were recorded and subtracted from all sample spectra afterwards in a self-written Matlab script. Additionally, the spectra of pure liposomes and DA were subtracted from the spectra of the mixtures with XSA. The corrected spectra were fitted and quantitatively analyzed with the open software BeStSel.[4]

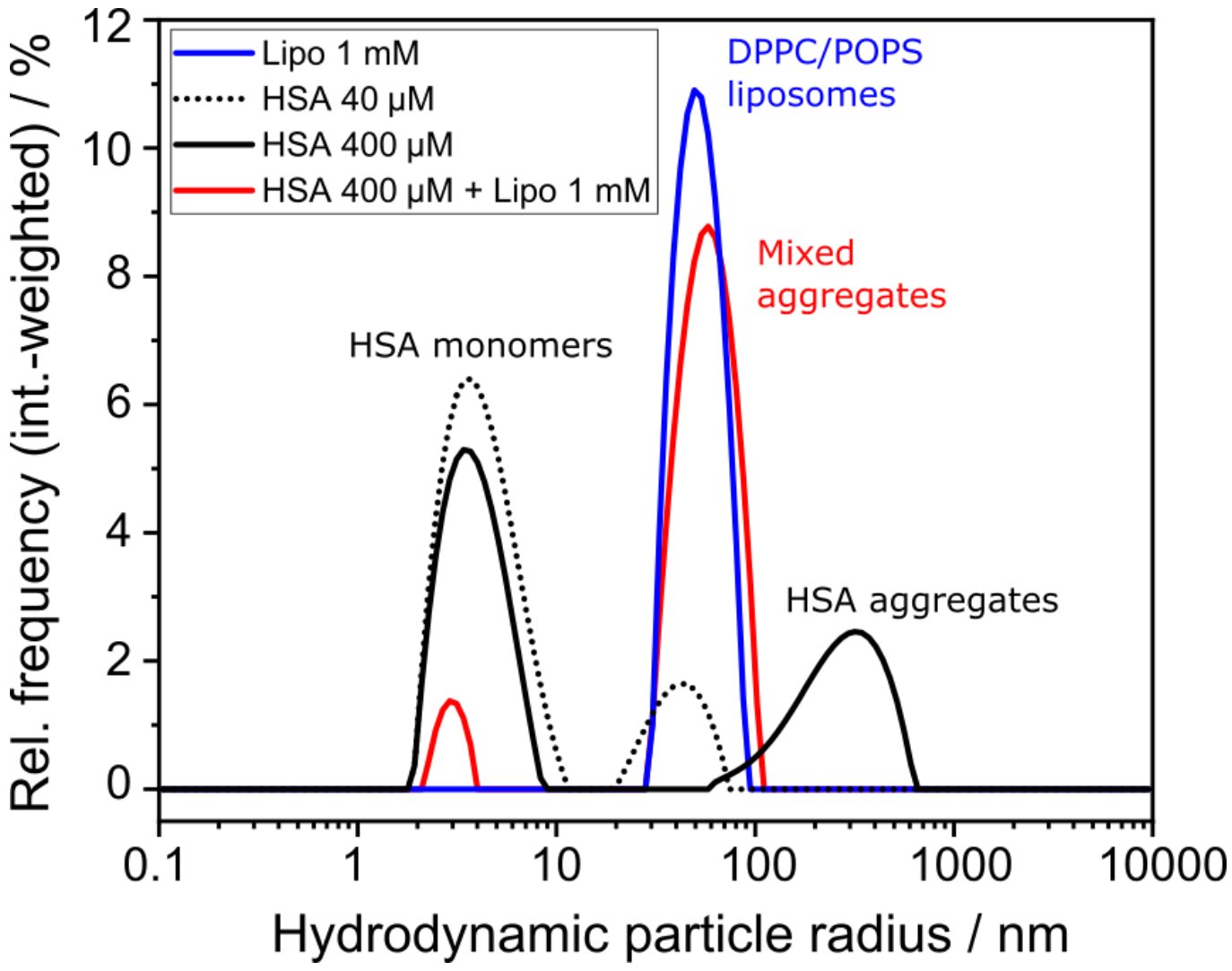

Figure S 1: DLS particle radius distributions of DPPC/POPS liposomes (1 mM) with HSA (40/400 µM) at pH 7.4

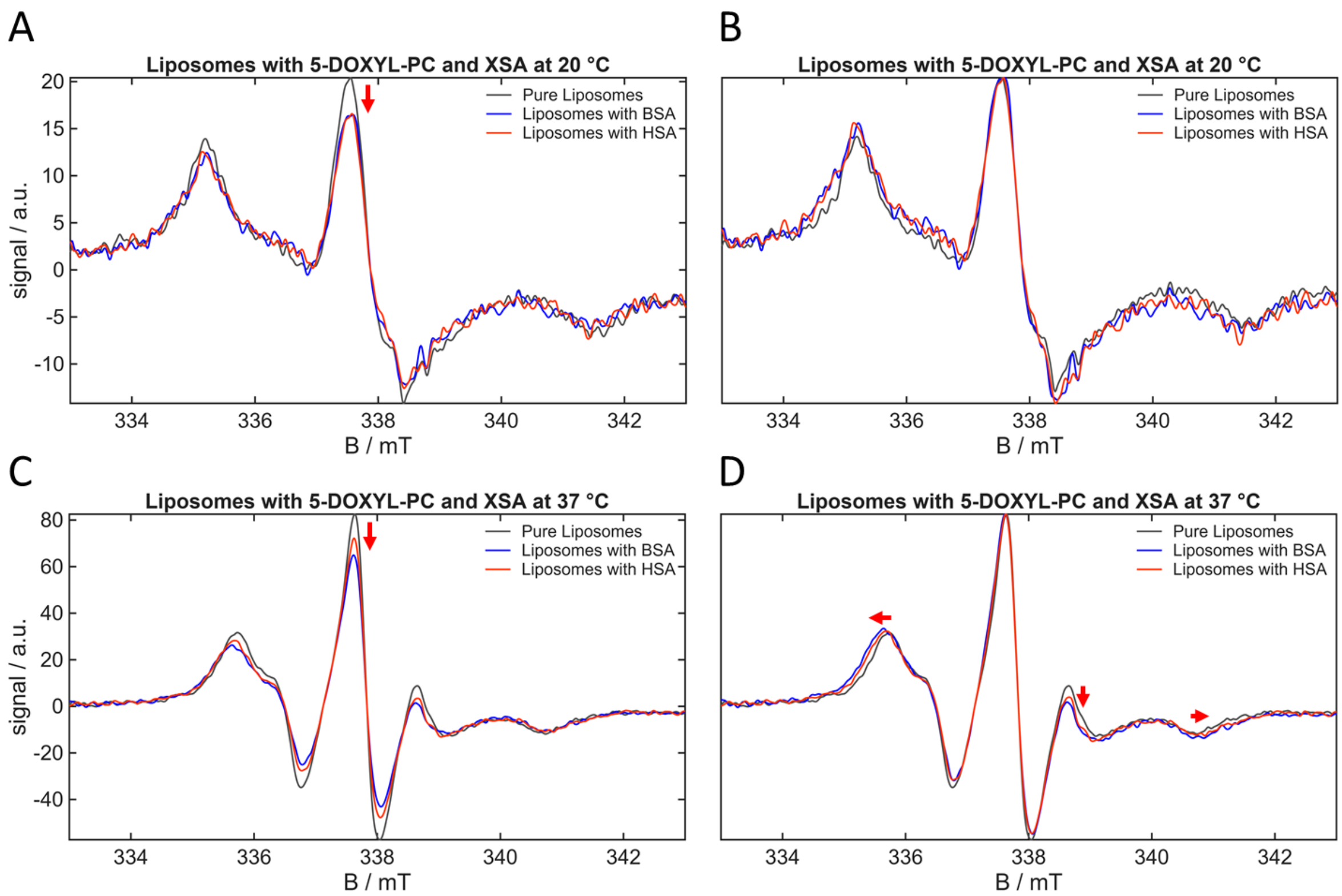

Figure S 2: CW EPR spectra of DPPC/POPS liposomes (80/20 mol-%) 1 mM with 5-DOXYL-PC 100 µM. Non-normalized (A, C) and intensity-normalized spectra (B, D) of pure liposomes and in mixtures with 400 µM BSA or HSA are shown. The spectra were measured at 20 °C (A, B) and 37 °C (C, D). Red arrows indicate characteristic changes of the spectral species.

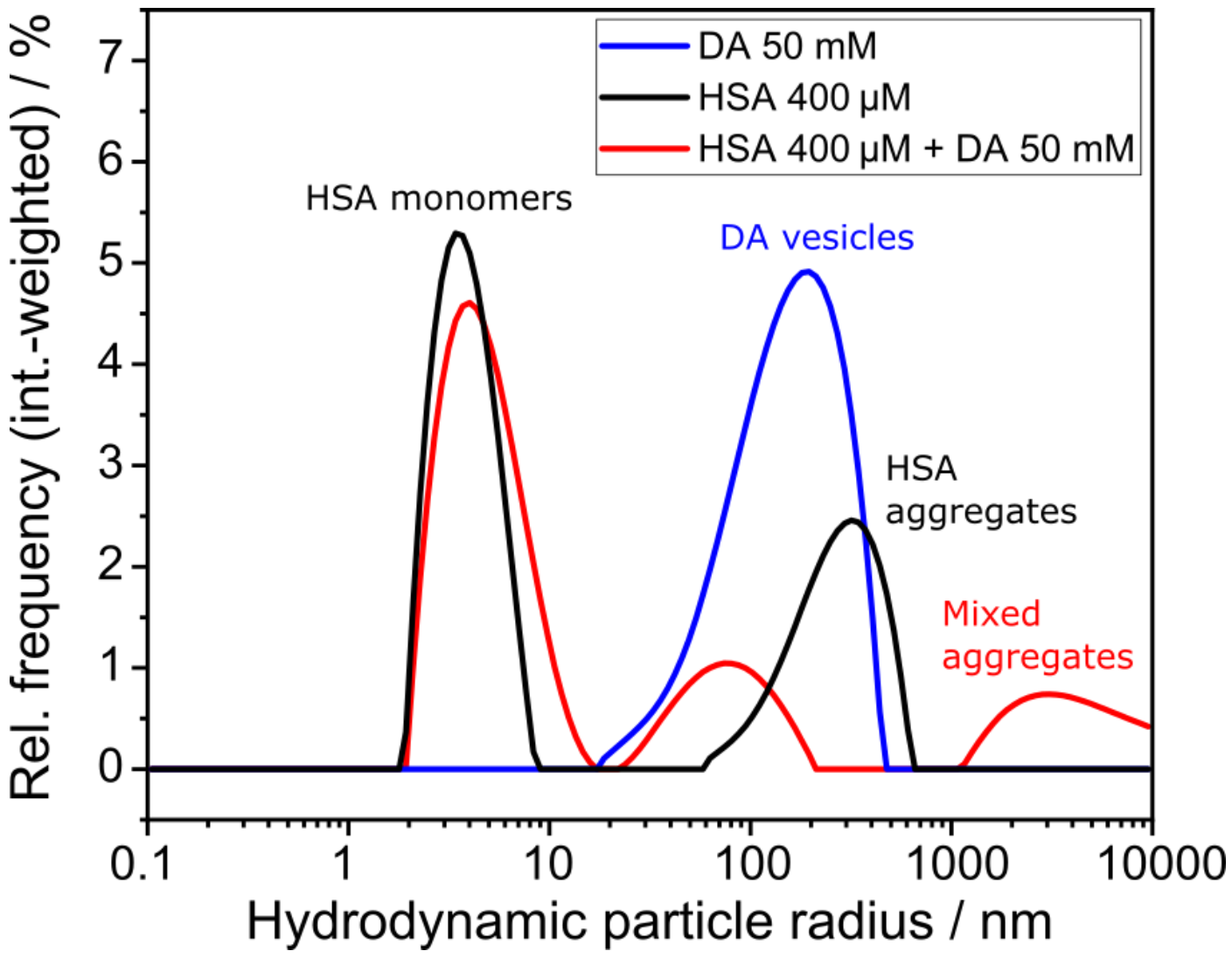

Figure S 3: DLS particle radius distributions of DA vesicles (50 mM) with 400 µM HSA at pH 7.4.

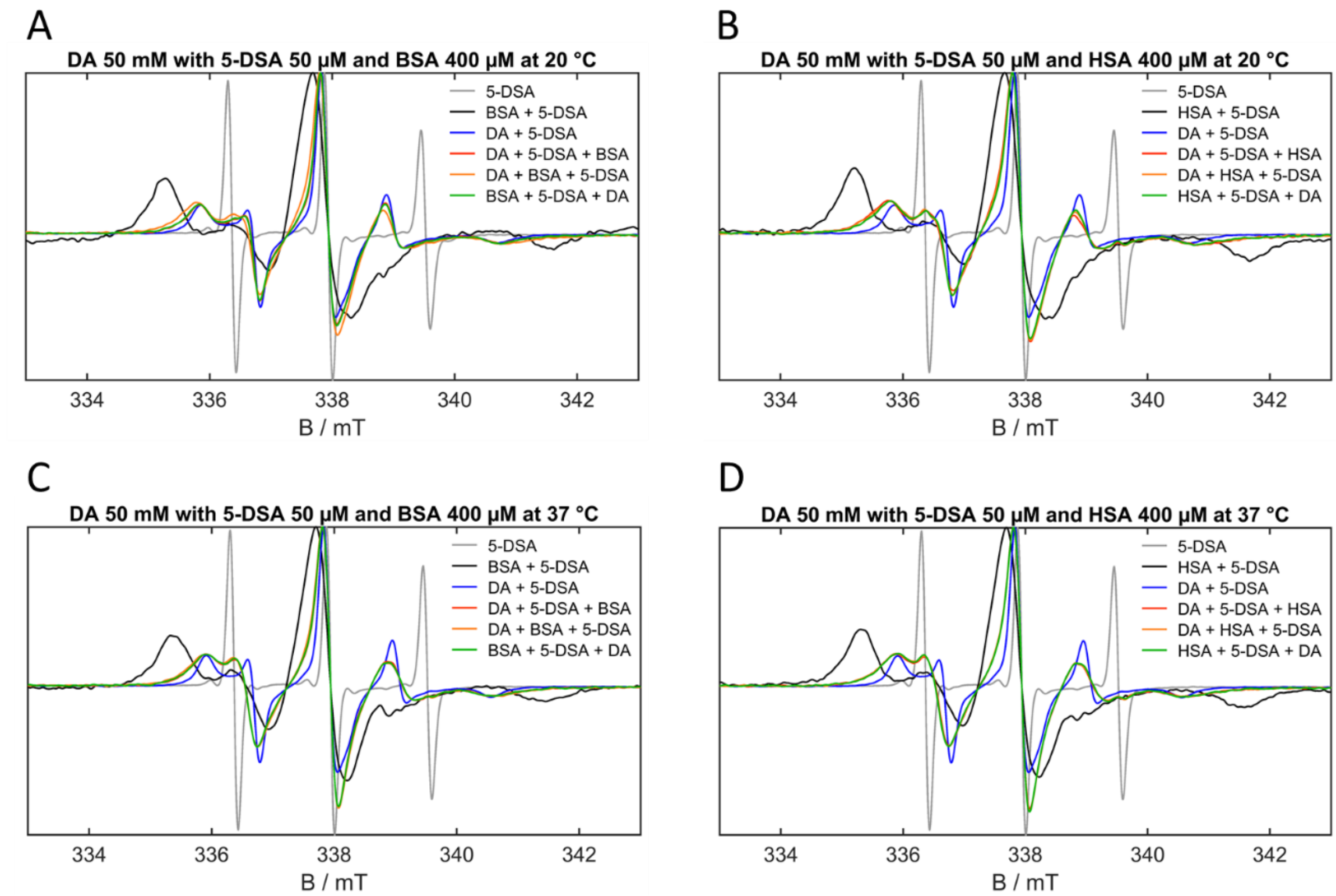

Figure S 4: CW EPR spectra of DA aggregates mixed with 5-DSA and XSA prepared in different mixing sequences of the components (see legend, entry sequence = mixing sequence). (A) CW EPR spectra with BSA at 20 °C. (B) CW EPR spectra with HSA at 20 °C. (C) CW EPR spectra with BSA at 37 °C. (D) CW EPR spectra with HSA at 37 °C. All spectra were normalized on the maximum intensity. All samples were set to pH 7.4.

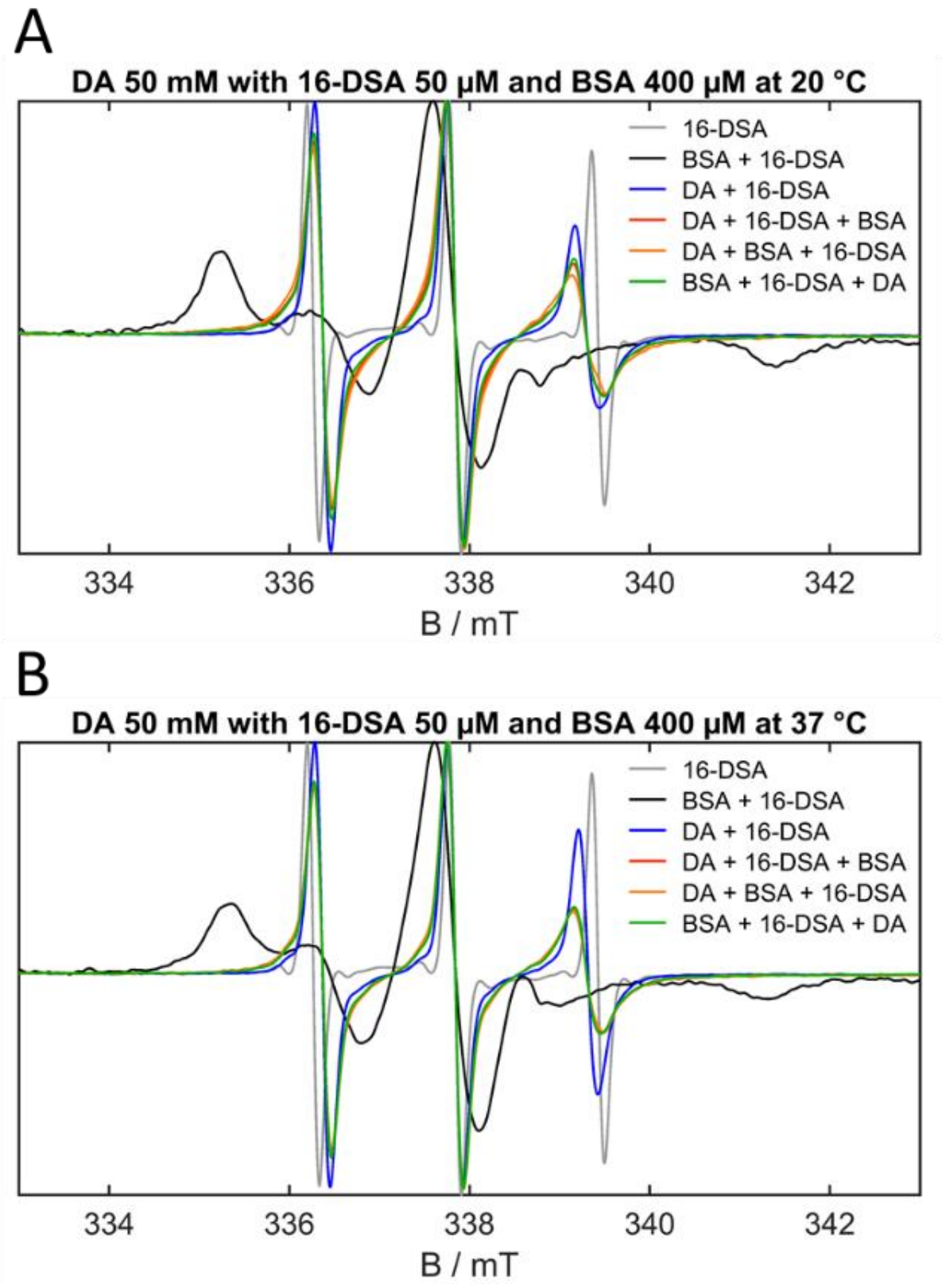

Figure S 5: CW EPR spectra of DA aggregates mixed with 16-DSA and BSA in different mixing sequences of the components (see legend, entry row = mixing row). (A) CW EPR spectra at 20 °C. (B) CW EPR spectra at 37 °C. All samples were set to pH 7.4. The effect of BSA is less pronounced when using 16-DSA as spin probe compared to 5-DSA, hence, the measurement series was not repeated with HSA and the main analysis focusses on 5-DSA instead. But a broadening effect of BSA is clearly visible and appears to be maximized when BSA and DA are pre-mixed (orange line).

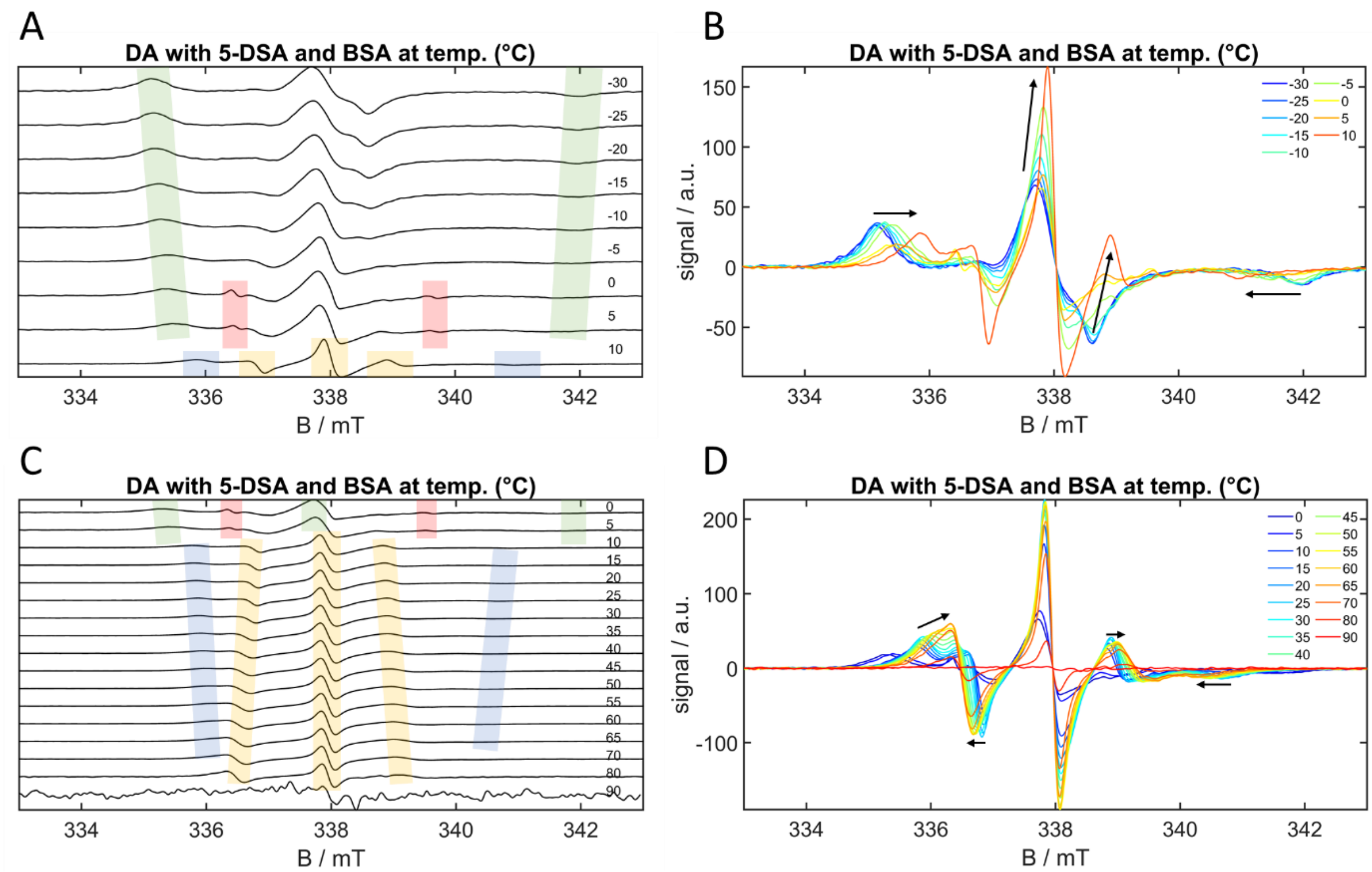

Figure S 6: Temperature dynamics of mixed DA/5-DSA aggregates in interaction with BSA. 50 mM DA were mixed with 50 µM 5-DSA and 400 µM BSA at pH 7.4. Depicted are stacked (A) and non-stacked (B) CW EPR spectra from -30 to 10 °C; and stacked (C) and non-stacked (D) spectra from 0 to 90 °C. Green bands mark the characteristic signals from immobilized 5-DSA which is bound to BSA (and frozen from -30 to -5 °C). Blue and yellow bands mark the signals arising from 5-DSA immobilized within DA aggregates as clearly distinguishable from albumin-binding by the "jump" of A_{zz} between the green and the blue band. Red bands mark free 5-DSA molecules which only appear at 0 and 5 °C.

Comment on the temperature dependence of DA/XSA mixtures:

Thawing of the solution at 0 °C can be clearly identified in the increase of the rotational dynamics of component (A) and the occurrence of component (F). From 10 to 90 °C a continuous transition within the spectra occurs, probably due to changes in the *A*- and the τ_c-tensor values. As it can be seen in **Figure S 6 C**, the "outer" signals of component (D) (blue bands) move to the center field, the radical dynamics increase. Simultaneously, the "inner" signals (yellow bands) drift outwards arguing for a more hydrophilic environment. Possibly, the aggregates are destabilized at higher temperatures and BSA re-binds the free ligands, or the protein actively solubilizes the DA aggregates, binding DA and 5-DSA competitively. At 80 °C a complete radical deactivation occurs within 8-10 min and no signal recovery occurs after that. Hence, the process is irreversible.

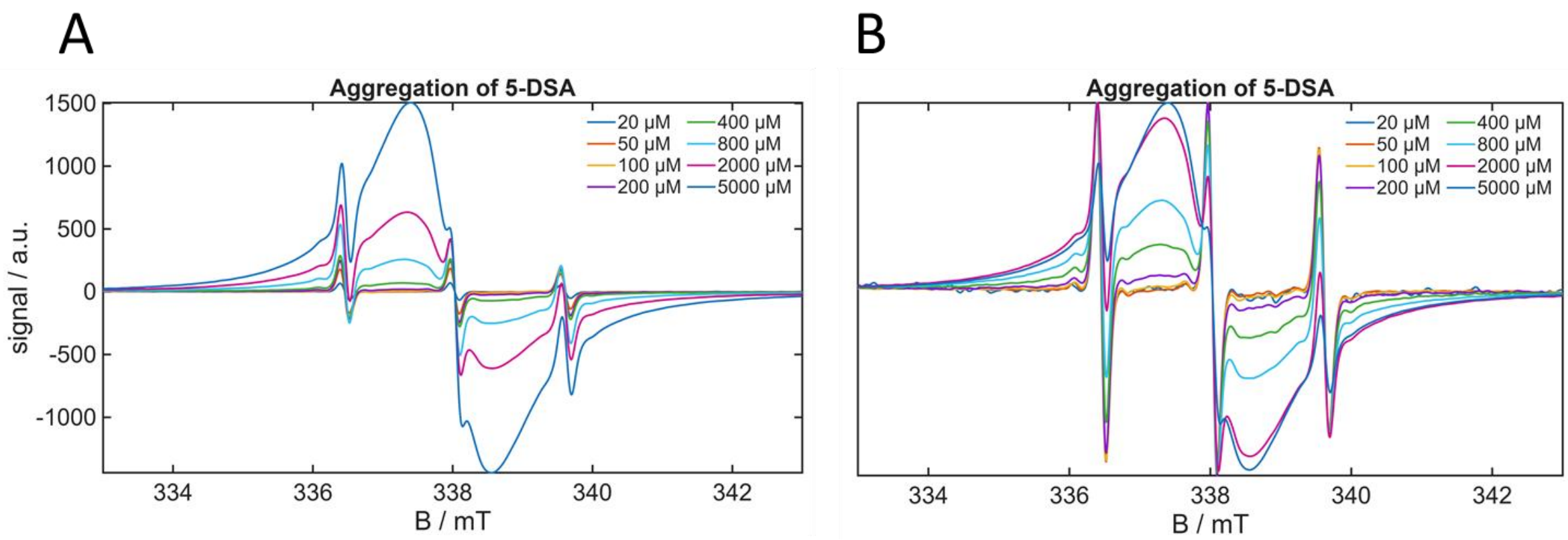

Figure S 7: Concentration-dependent aggregate formation of 5-DSA. (A) Non-normalized CW EPR spectra series. (B) Intensity-normalized CW EPR spectra series.

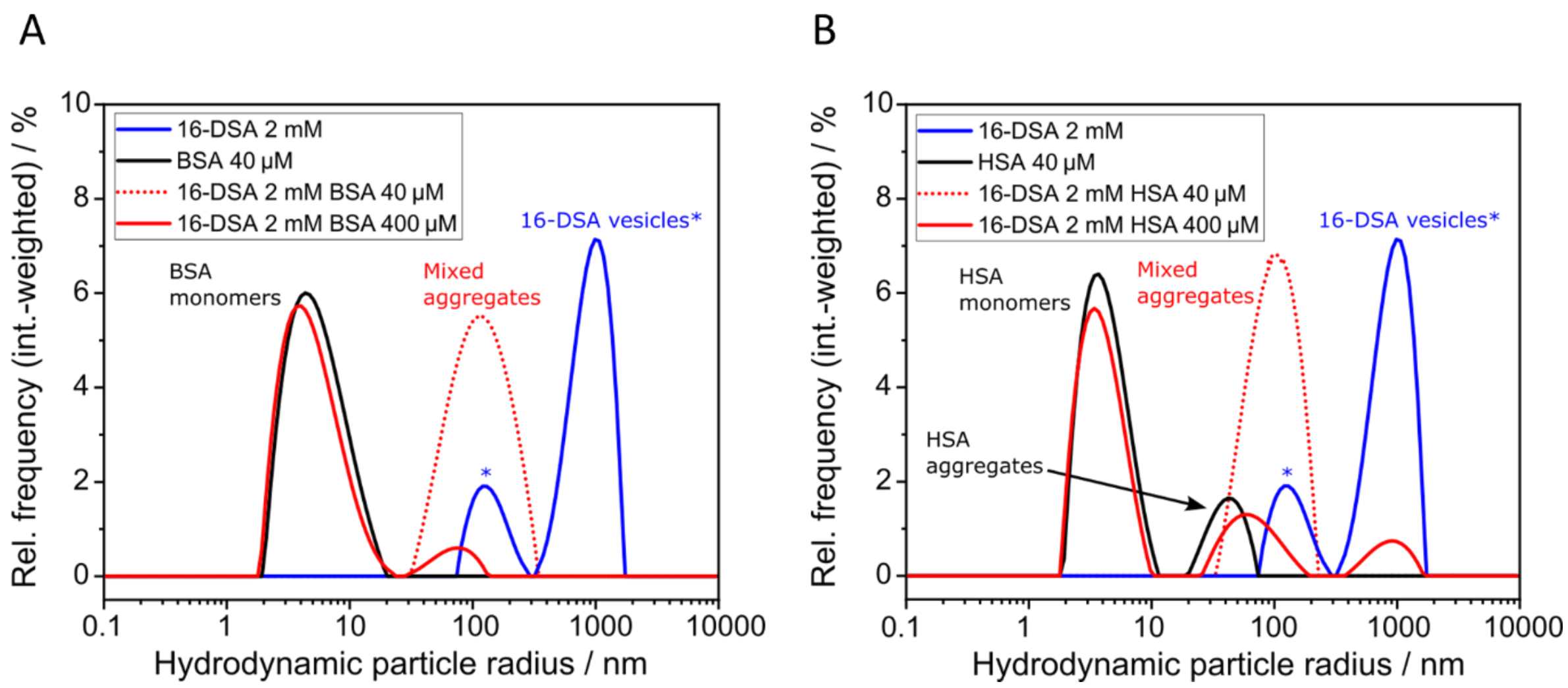

Figure S 8: DLS particle radius distributions of 16-DSA vesicles with BSA (A) and HSA (B) at pH 7.4.

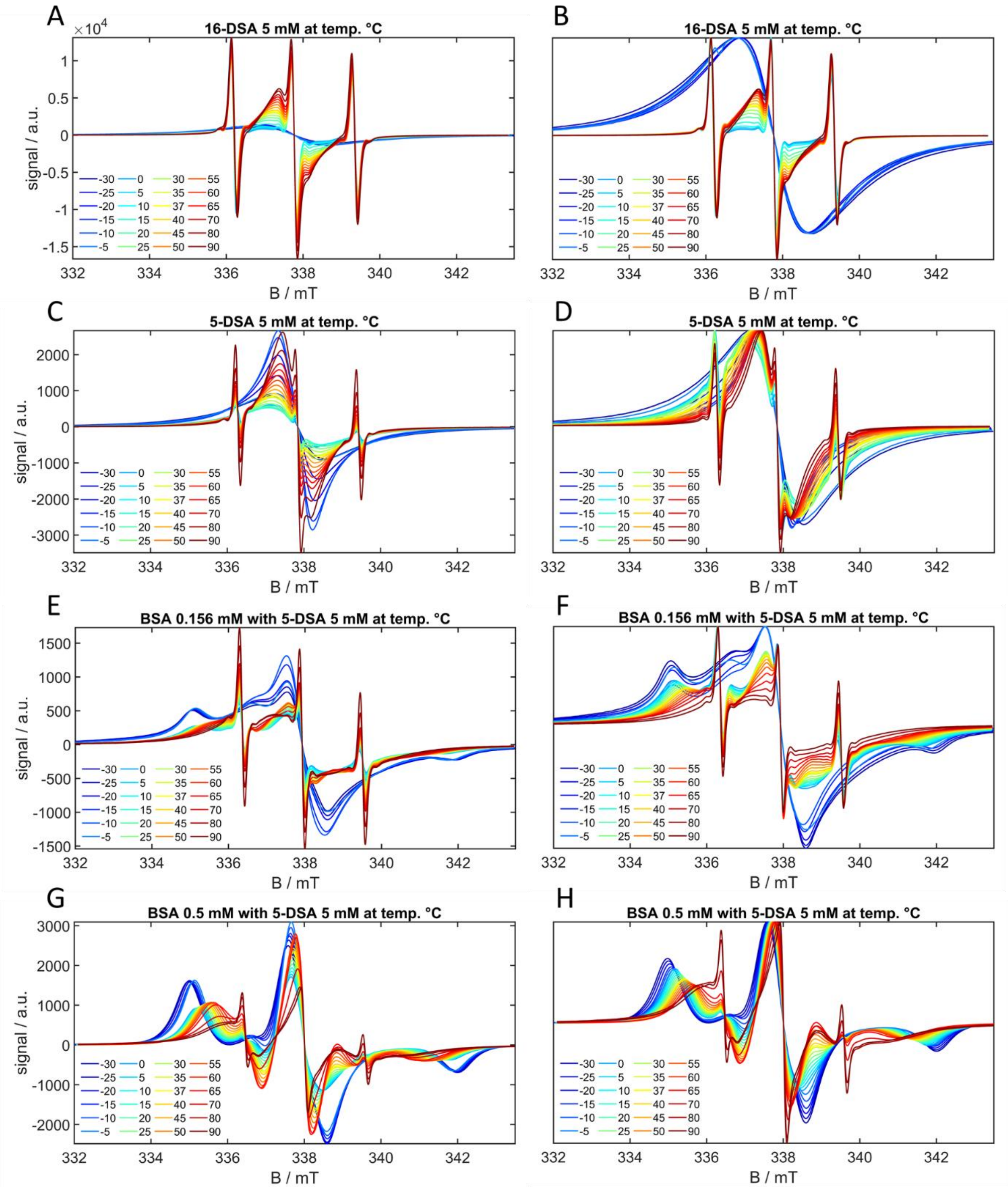

Figure S 9: Temperature dynamics in the CW EPR spectra of different aggregate-containing systems. A-B: 16-DSA aggregates at 5 mM with non-normalized and intensity-normalized spectra. C-D: 5-DSA aggregates at 5 mM with non-normalized and intensity-normalized spectra. E-F: 5-DSA 5 mM with BSA 0.156 mM. G-H: 5-DSA 5 mM with BSA 0.5 mM.

Comment on the temperature dynamics of 5/16-DSA aggregates:

As visualized in **Figure S 9A-B**, 16-DSA shows a very broad, aggregated component (E) at low temperatures which becomes at first sharper from -30 to -20 °C, arguing for a rising exchange frequency.

Then the signal becomes slightly broader at -15 °C and sharper again with rising temperature. The first small signal of free 16-DSA appears already at -5 °C, arguing for a freezing point reduction in the system. At 0 to 10 °C the proportion of (E) becomes extremely small (almost 0 %), while free 16-DSA dominates. Afterwards, (E) increases again at 20 °C and higher temperatures, with an increasing exchange frequency.

As can be seen in **Figure S 9C-D**, 5-DSA shows a dominant proportion of (E) at all temperatures but an interesting change of the exchange frequency as a function of the temperature, as well. (E) is very broad at -30 °C but becomes sharper at first from -30 to -10 °C. Afterwards, it becomes significantly broader again at -5 °C, and sharper again at 0 °C, at which signals of free 5-DSA (F) appear besides (E). Between 10 and 20 °C a notable intensity variation of component (E) with a decrease and increase is visible. This might signalize a phase transition of the 5-DSA vesicles. From 20 to 90 °C, a small proportional increase of (F) is visible while (E) becomes sharper and all components become more dynamic. The exchange frequency of (E) increases with rising temperature, like in the case of 16-DSA.

In the mixture with 156 µM BSA, the spectra of 5-DSA in **Figure S 9E-F** show a complex superposition of (E), (A) and (F). Especially at -5 °C the co-existing components (E) and (A) are well visible, while (F) is not yet appearing. With increasing temperature, the proportion of (E) seems to decrease compared to (A) and (F). This observation clearly shows that the triangle equilibrium is strongly temperature-dependent, since the cac of FA aggregates and the binding affinity of albumin towards FAs are also supposed to be temperature-dependent. The latter can be seen in the temperature series of BSA with 5-DSA in **G-H** and is already known.[5]

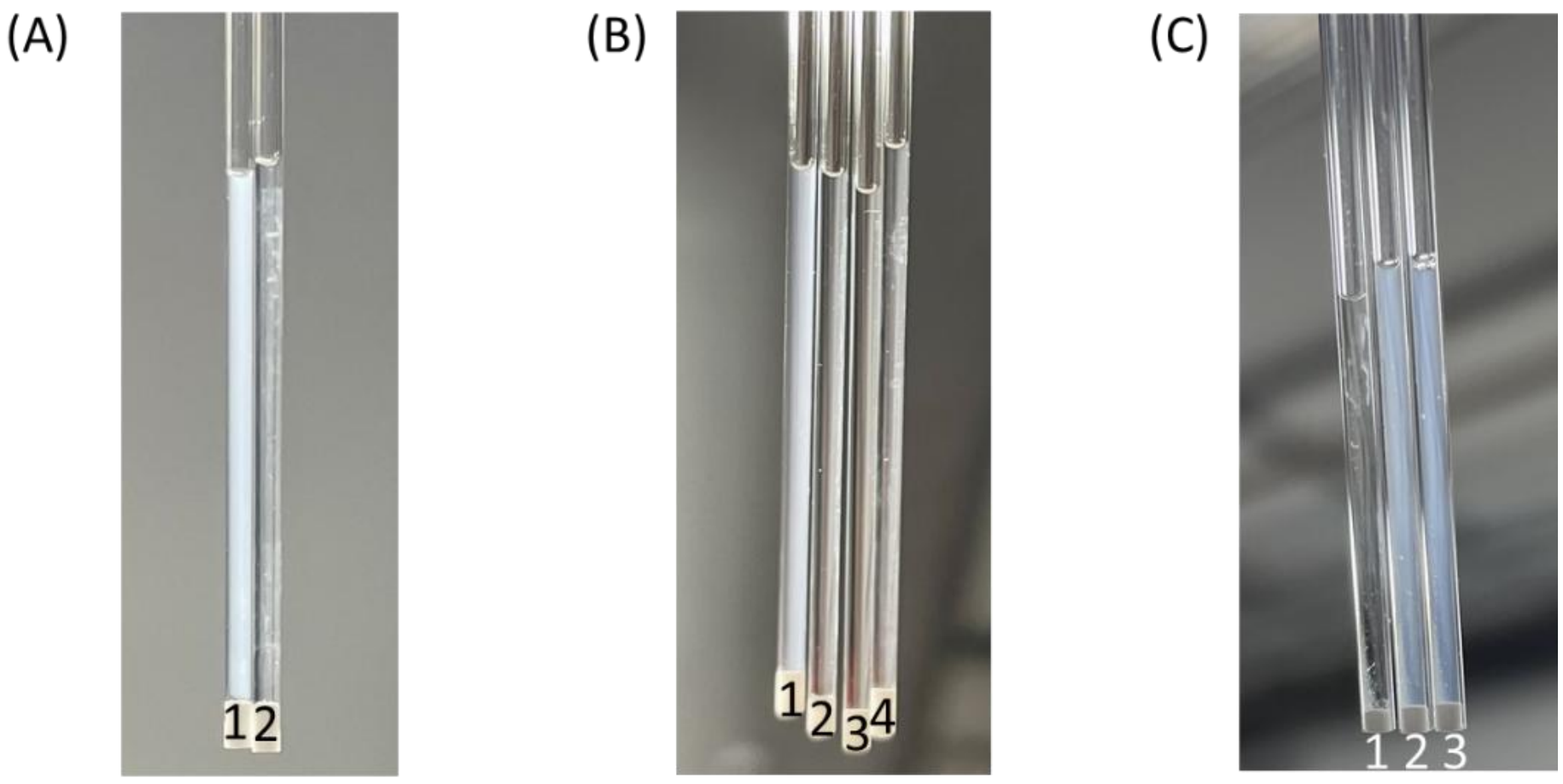

Figure S 10: Turbidity comparison of different aggregate/BSA-containing solutions filled into EPR capillaries. (A-1) 50 mM DA. (A-2) 50 mM DA with 400 µM BSA. (B-1) 50 mM DA. (B-2,3,4) 50 mM DA with 50 µM 16-DSA and 400 µM BSA in different sequences. (C-1) 700 µM BSA with 5 mM 16-DSA. (C-2) 300 µM BSA with 5 mM 16-DSA. (C-3) 5 mM 16-DSA.

Table S 1: Detected light transmittance during DLS measurements for various samples.

Sample	DLS light transmittance / %
BSA 400 µM 16-DSA 2 mM	99.7
HSA 400 µM 16-DSA 2 mM	100
BSA 40 µM 16-DSA 2 mM	**65**
HSA 40 µM 16-DSA 2 mM	**71.3**
16-DSA 2 mM	**21.6**
BSA 400 µM 5-DSA 2 mM	96.5
HSA 400 µM 5-DSA 2 mM	100
BSA 40 µM 5-DSA 2 mM	100
HSA 40 µM 5-DSA 2 mM	100
5-DSA 2 mM	100
BSA 400 µM	99.2
HSA 400 µM	100
BSA 40 µM	100
HSA 40 µM	100

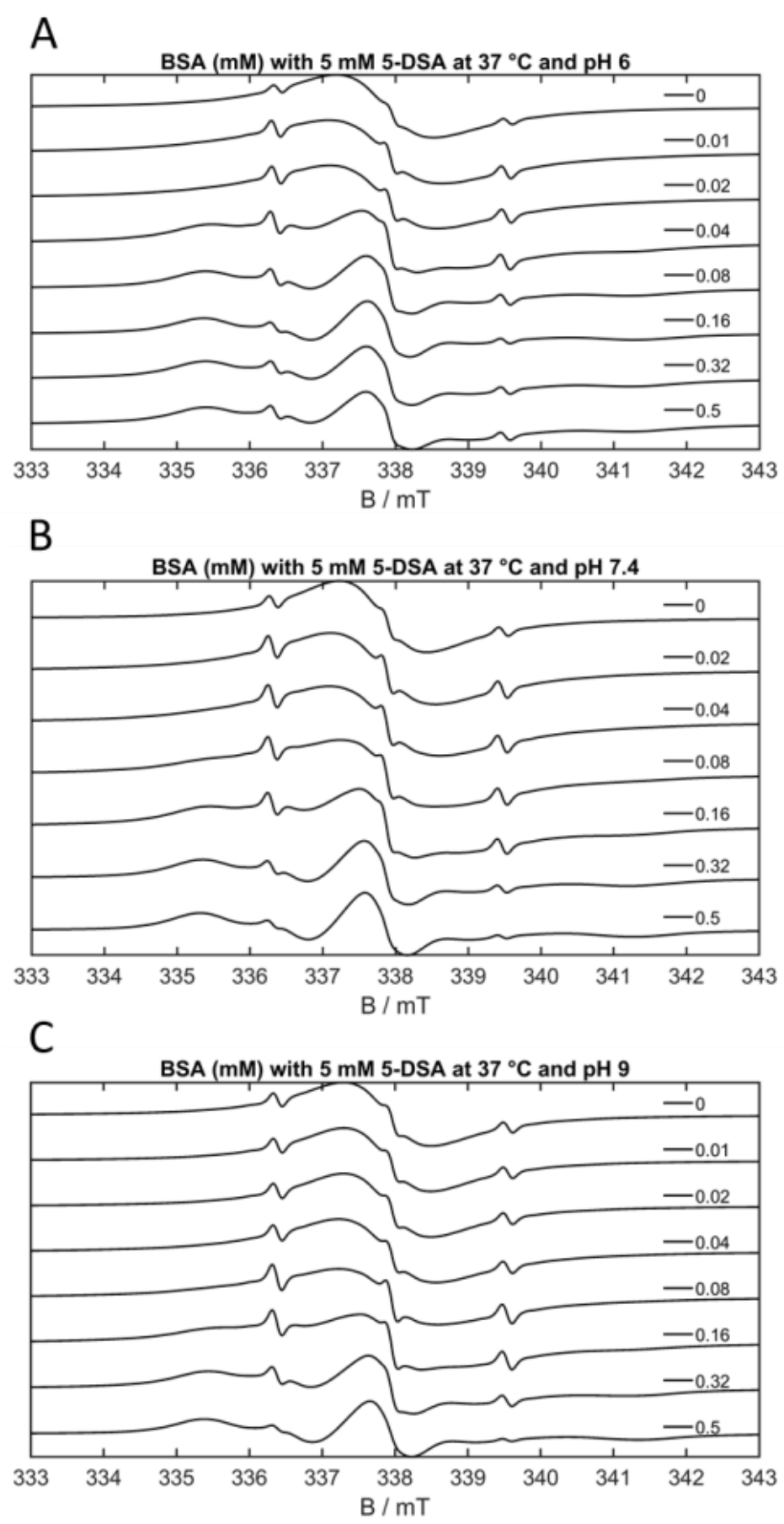

Figure S 11: pH-dependent CW EPR concentration series of BSA with 5 mM 5-DSA. (A) BSA with 5-DSA at pH = 6. (B) BSA with 5-DSA at pH = 7.4. Spectra in (B) are identical to the ones in **Figure 4**. (C) BSA with 5-DSA at pH = 9.

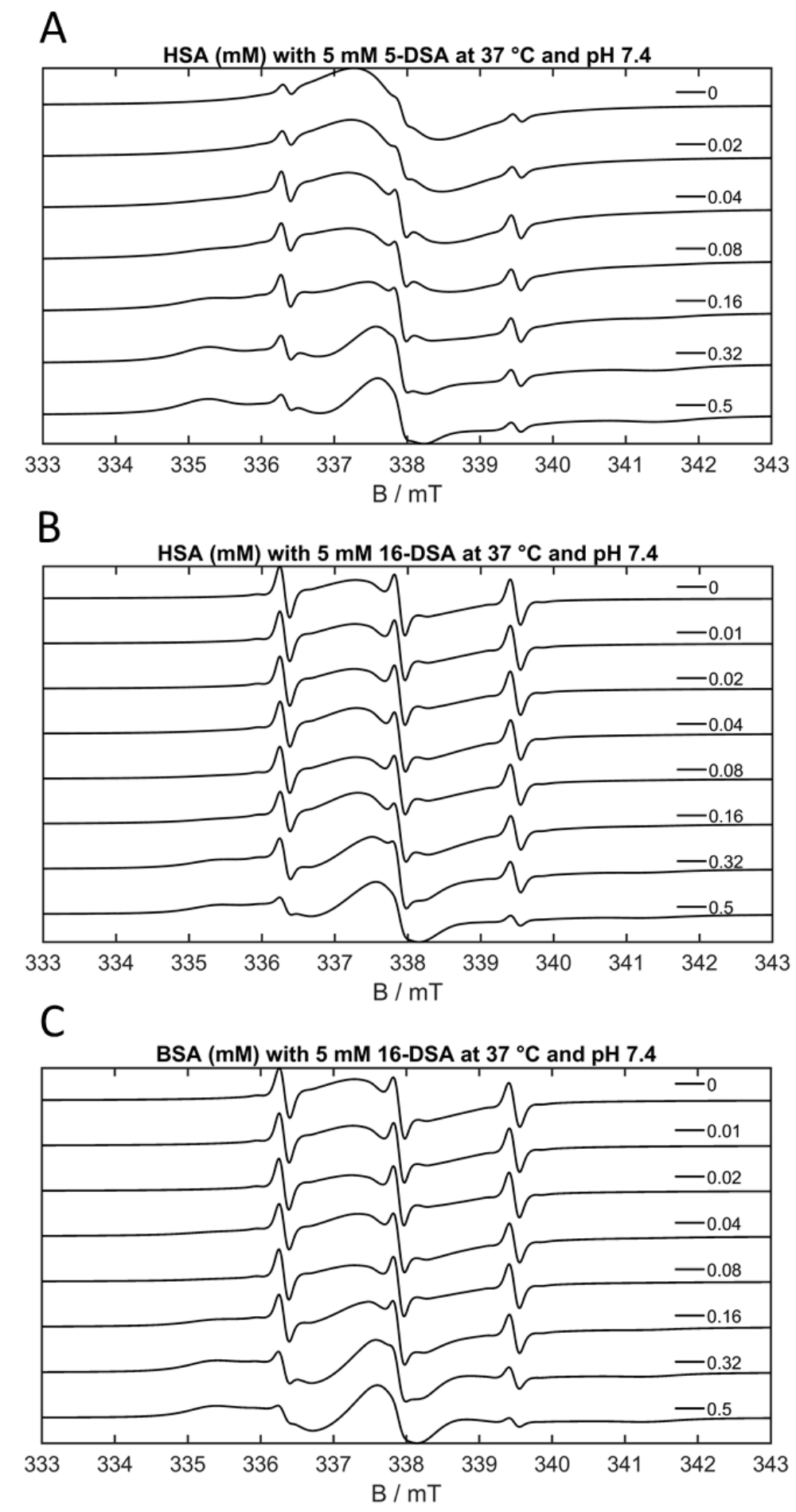

Figure S 12: CW EPR spectra of concentration series of HSA with (A) 5 mM 5-DSA and (B) 5 mM 16-DSA, as well as (C) BSA with 5 mM 16-DSA. Spectra in (A) are identical to the ones in **Figure 4**.

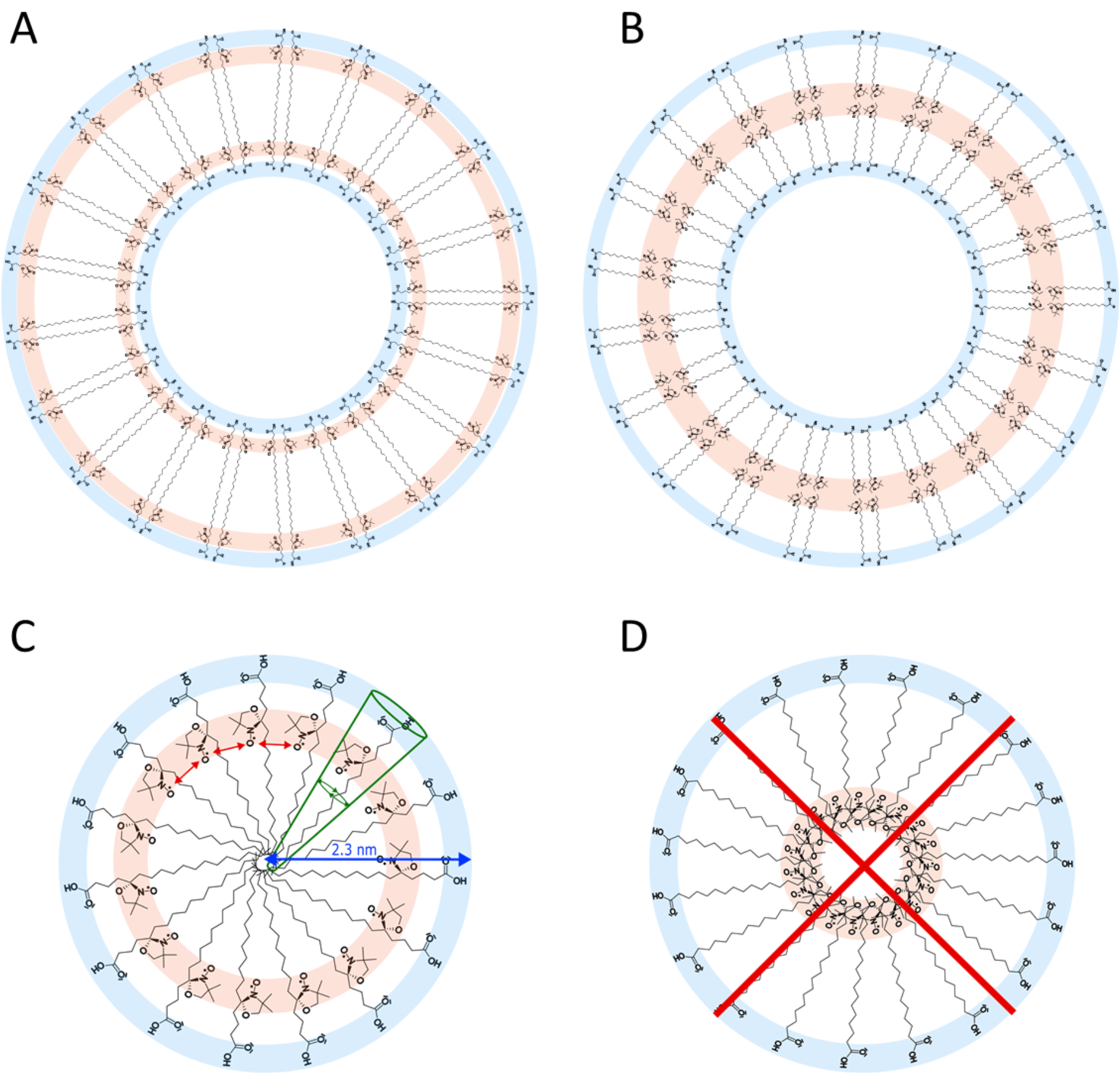

Figure S 13: Simplified schemes of theoretical, spherical micellar or vesicular structures of 5- and 16-DSA monomers and hydrogen-bonded dimers. Note that the FAs are drawn in a linear conformation while in reality back-folding might occur. (A) Spherical vesicle formed by 5-DSA. (B) Spherical vesicle formed by 16-DSA. The local concentration of DOXYL groups is higher in this case, leading to a higher exchange frequency. (C) Spherical micelle of 5-DSA with an assumed conical geometry of the monomer. Linearly stretched 5-DSA has a length of approximately 2.3 nm. Considering effects like an enlargement of the sphere due to the packing density and electrostatic repulsion, an ellipsoidal deformation and the aqueous layer beyond the FA headgroups, a hydrodynamic micelle radius between 3 and 10 nm seems to be realistic. (D) The bulky DOXYL group at the end of the chain is expected to prevent the formation of 16-DSA micelles.

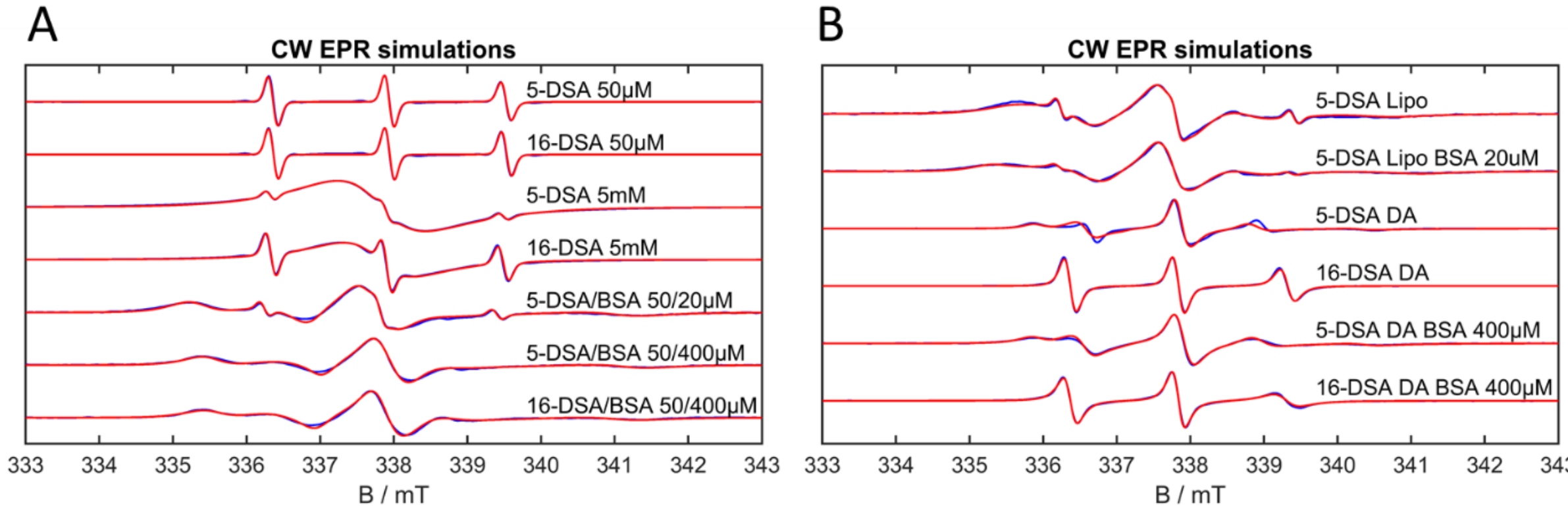

Figure S 14: CW EPR simulations (red) and spectra (blue) of different systems at 37 °C. (A) 5/16-DSA at 50 µM and 5 mM, 5-DSA with BSA in the ratios 50/20 µM and 50/400 µM, 16-DSA with BSA 50/400 µM. (B) 50 µM 5-DSA with 1 mM DPPC/POPS liposomes pure and with 20 µM BSA, 50 µM 5/16-DSA with 50 mM DA pure and with 400 µM BSA.

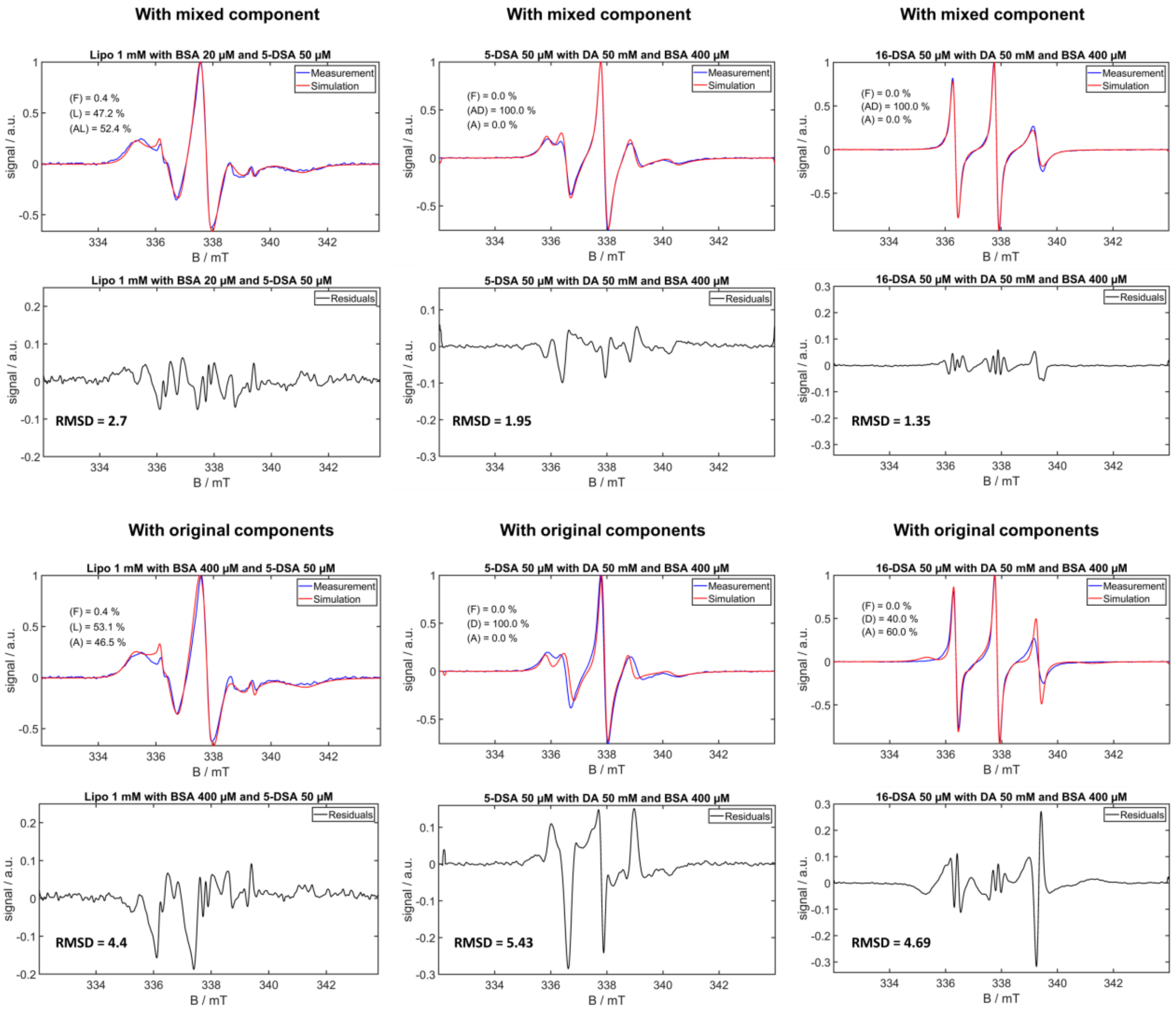

Figure S 15: Comparison of CW EPR simulations with the original pure components and the mixed components AL and AD for three example systems. AD refers to the mixed component for 5-DSA or 16-DSA in the respective example. Top rows: simulations and residuals with mixed components. Bottom rows: simulations and residuals with the pure, superimposed components. Component proportions were fitted to generate a simulation with a minimum RMSD value between simulated and measured spectrum. The comparison of the simulations and the residuals validates the existence of the components AL and AD. In case of 5-DSA with DA and BSA the addition of (A) led in any case to an increase of the residuals.

Comment on the simulations of mixed components: The residuals of the simulations compared to the measured spectra were plotted for three examples (liposome with 5-DSA and BSA, DA with 5-DSA and BSA, DA with 16-DSA and BSA), in each case with the optimum simulation including the mixed components (AL) or (AD) (5/16-DSA) on the top and with an alternative simulation including only the original pure components A and L or D as a superposition in the lower part. Their basic simulation parameters were not changed and only their proportions were adjusted to achieve the best RMSD values. The resulting RMSD values and the residual plots demonstrate very well that in each of the three cases the use of the mixed component leads to a drastic improvement of the quality for the simulated spectra, a reduced RMSD value and smaller residuals compared to the simulations in which the pure components were used instead. In case of (AD) with 5-DSA, the best fit of the superposition simulation requires 0% (A) and 100% (D), since every addition of (A) increases the RMSD value, while in case of (AD) with

16-DSA the addition of (A) improves the RMSD value slightly but leads to unrealistic spectral features stemming from the albumin-bound component which are also visible in the residual plot. Proportions of free components in the liposome example were not varied, only the proportions of component (L), (A) and (AL) were varied between the two cases.

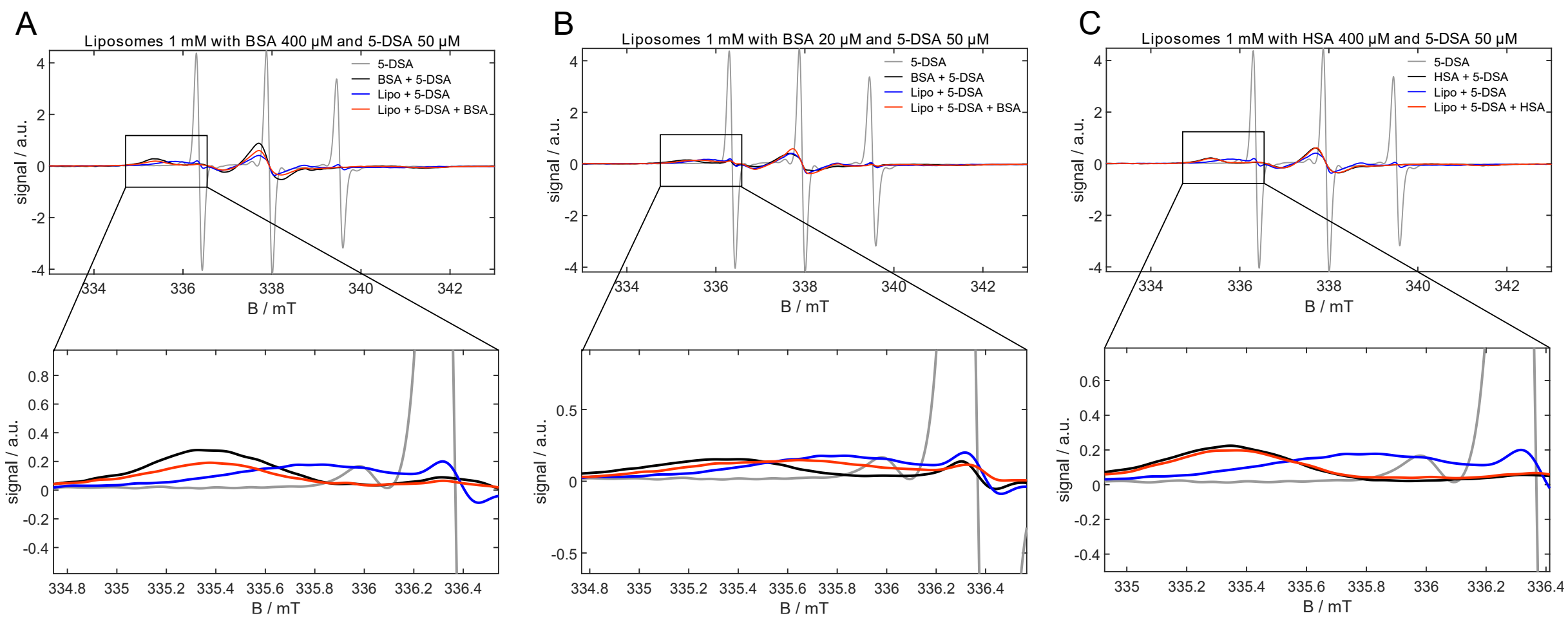

Figure S 16: Alternative visualization of the liposome-related CW EPR spectra from **Figure 2**. The spectra were here normalized on their double integrals and zoomed insets below show the low-field areas with the strongest deviations between the spectral components.

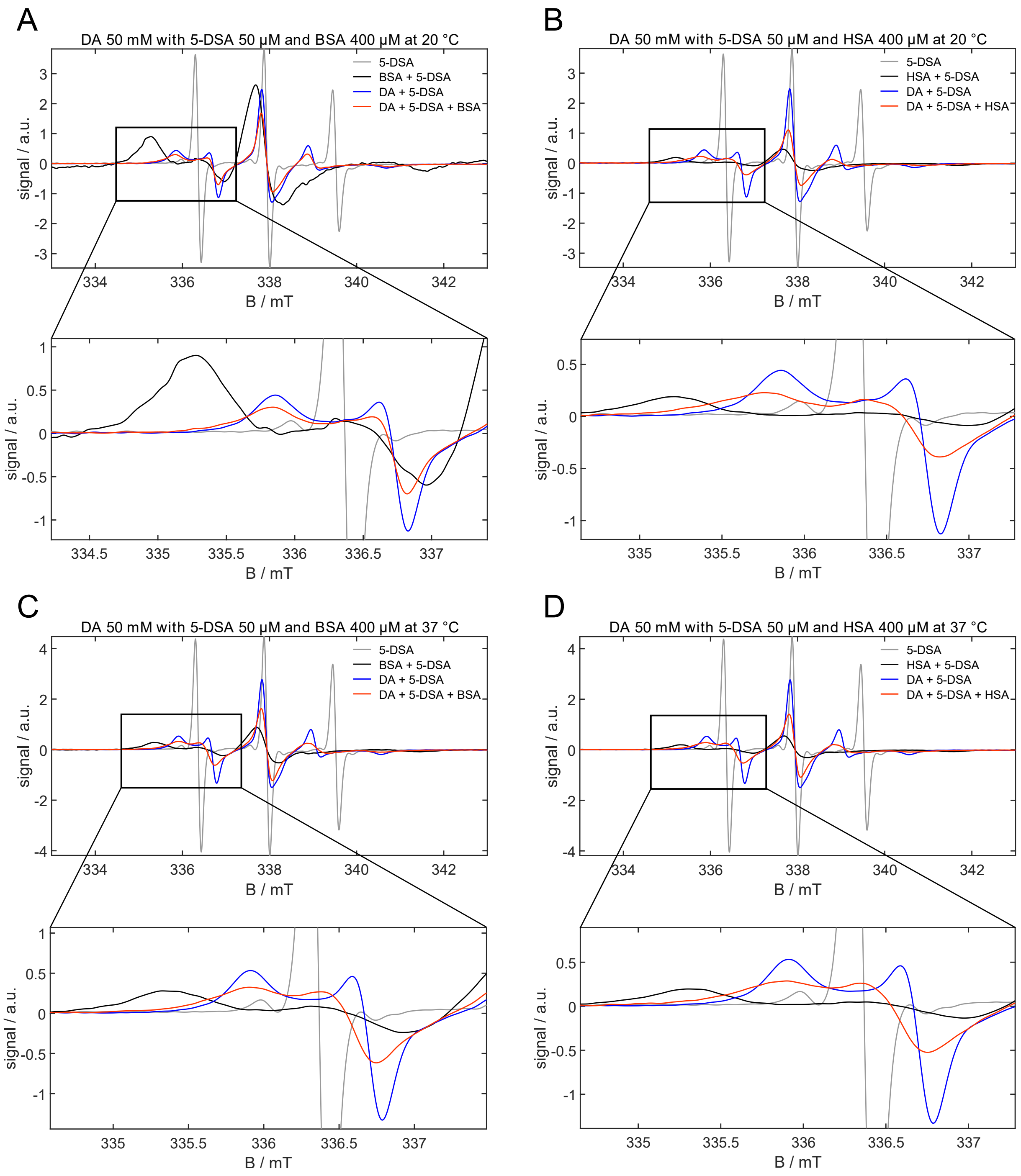

Figure S 17: Alternative visualization of the DA-related CW EPR spectra from **Figure 3**. The spectra were here normalized on their double integrals and zoomed insets below show the low-field areas with the strongest deviations between the spectral components.

Table S 2: Simulated parameters of different spectral components in samples measured at pH 7.4 and 37 °C. A = ^{14}N-hyperfine coupling tensor value, decreasing values indicate less polar molecular environments. τ_c = rotational correlation time tensor values, increasing values indicate stronger interactions. ν_{ex} = Heisenberg exchange frequency, higher values originate from higher local radical concentrations. $Lw_{g,l}$ = gaussian/lorentzian linewidths. α, β, γ = Euler angles for the diffusion tensor.

Comp.	A_{xx}/MHz	A_{yy}/MHz	A_{zz}/MHz	$\tau_{c,xx}$ /ns	$\tau_{c,yy}$ /ns	$\tau_{c,zz}$ /ns	ν_{ex} /MHz	$Lw_{g,l}$	α, β, γ/rad
$F_{5\text{-DSA}}$	15.15	13.63	104.05	0.192	0.067	0.067	0	0.132, 0	0, 50, 0
$F_{16\text{-DSA}}$	15.15	13.63	104.05	0.161	0.056	0.056	0	0.14, 0	0, 50, 0
$A_{5\text{-DSA}}$	15	17.5	95	39.1	44.9	3.39	0	0, 0.12	0, 49, 0
$A_{16\text{-DSA}}$	16	19	95.5	39.1	22.51	2.69	0	0, 0.07	0, 49, 0
$L_{5\text{-DSA}}$	18.4	20	88.4	20.02	9.37	1.22	5	0.12, 0.01	0, 33, 0
$AL_{5\text{-DSA}}$	15	17.5	93	61.94	71.1	3.39	0	0, 0.17	0, 49, 0
$E_{5\text{-DSA}}$	15.15	12.93	103.18	0.241	0.085	0.198	59	0, 0.2	0, 32, 0
$E_{16\text{-DSA}}$	15.15	12.93	102.18	0.242	0.067	0.134	65	0, 0.01	0, 35, 0
$D_{5\text{-DSA}}$	14	27	81	1.7	125.8	157.5	0	0.04, 0.04	0, 55, 0
$D_{16\text{-DSA}}$	14.15	13.73	96	0.762	0.211	0.168	0	0.11, 0.1	0, 50, 0
$AD_{5\text{-DSA}}$	16	28	82	1.7	125.8	157.5	0	0.08, 0.13	0, 55, 0
$AD_{16\text{-DSA}}$	14.15	13.73	96.1	2.23	0.82	0.653	0	0.03, 0.04	0, 50, 0

Table S 3: Apparent isotropic hyperfine coupling constants and rotational correlation times of all spectral components.

Comp.	a_{iso}’	$\tau_{c,\ iso}$’
$F_{5\text{-DSA}}$	44.28	0.109
$F_{16\text{-DSA}}$	44.28	0.091
$A_{5\text{-DSA}}$	42.5	29.13
$A_{16\text{-DSA}}$	43.5	21.43
$L_{5\text{-DSA}}$	42.27	10.2
$AL_{5\text{-DSA}}$	41.83	45.48
$E_{5\text{-DSA}}$	43.75	0.175
$E_{16\text{-DSA}}$	43.42	0.148
$D_{5\text{-DSA}}$	40.67	95
$D_{16\text{-DSA}}$	41.29	0.38
$AD_{5\text{-DSA}}$	42	95
$AD_{16\text{-DSA}}$	41.33	1.23

Table S 4: Component proportions Φ of selected samples with mixtures of spectral species, measured at 37 °C. The root mean square deviations (RMSD) serve as error estimations. Note that (D) is a different component for 5-DSA and 16-DSA.

System	Φ (F)<br>%	Φ (A)<br>%	Φ (L)<br>%	Φ (AL)<br>%	Φ (D)<br>%	Φ (AD)<br>%	Φ (E)<br>%	RMSD<br>%
5-DSA 5 mM	0.398	-	-	-	-	-	99.6	1.87
16-DSA 5 mM	3.66	-	-	-	-	-	96.34	2.19
5-DSA 50 µM	100	-	-	-	-	-	0	1.66
16-DSA 50 µM	100	-	-	-	-	-	0	1.55
BSA 5-DSA 400/50	0	100	-	-	-	-	-	2.96
BSA 16-DSA 400/50	0	100	-	-	-	-	-	2.88
BSA 5-DSA 20/50	0.87	99.13	-	-	-	-	-	3.68
Lipo 5-DSA	1.1	-	98.9	-	-	-	-	3.27
Lipo 5-DSA BSA 20	0.39	-	44.27	55.34	-	-	-	2.67
DA 5-DSA	0	-	-	-	100	-	-	3.89
DA 16-DSA	0	-	-	-	100	-	-	1.35
DA 5-DSA BSA	0	0	-	-	0	100	-	1.95
DA 16-DSA BSA	0	0	-	-	0	100	-	1.35

Table S 5: Simulated component proportions, linewidths and exchange frequencies for the concentration series of BSA with 5 mM 5-DSA.

c(BSA) / µM	Φ (F) / %	Φ (E) / %	Φ (A) / %	$Lw_{g,l}$ (E)	ν_{ex} /MHz (E)	RMSD / %
0	0.398	99.60	0	0, 0.2	59	1.88
20	0.547	99.45	0	0.2, 0.21	49	3.52
40	0.531	96.57	2.90	0.5, 0.21	47	4.51
80	0.452	89.29	10.26	0.55, 0.2	46	4.94
160	0.387	77.48	22.14	0.55, 0.2	41	5.97
320	0.349	49.83	49.83	0.55, 0.2	34	5.10
500	0.279	19.94	79.78	0.55, 0.2	30	4.57

Table S 6: Simulated component proportions, linewidths and exchange frequencies for the concentration series of HSA with 5 mM 5-DSA. Deviations of the values for 0 mM compared to the same system in **Table S 5** occur due to the experimental error but can be neglected.

c(HSA) / µM	Φ (F) / %	Φ (E) / %	Φ (A) / %	$Lw_{g,l}$ (E)	ν_{ex} /MHz (E)	RMSD / %
0	0.349	99.65	0	0, 0.2	60	1.74
20	0.448	99.55	0	0.2, 0	52	2.85
40	0.786	98.23	0.98	0.2, 0	47	4.68
80	0.621	93.17	6.21	0.3, 0.14	44	4.72
160	0.701	77.88	21.42	0.3, 0.14	43	4.27
320	0.498	47.76	51.74	0.3, 0.18	39	3.55
500	0.352	35.17	64.48	0.3, 0.2	39	2.99

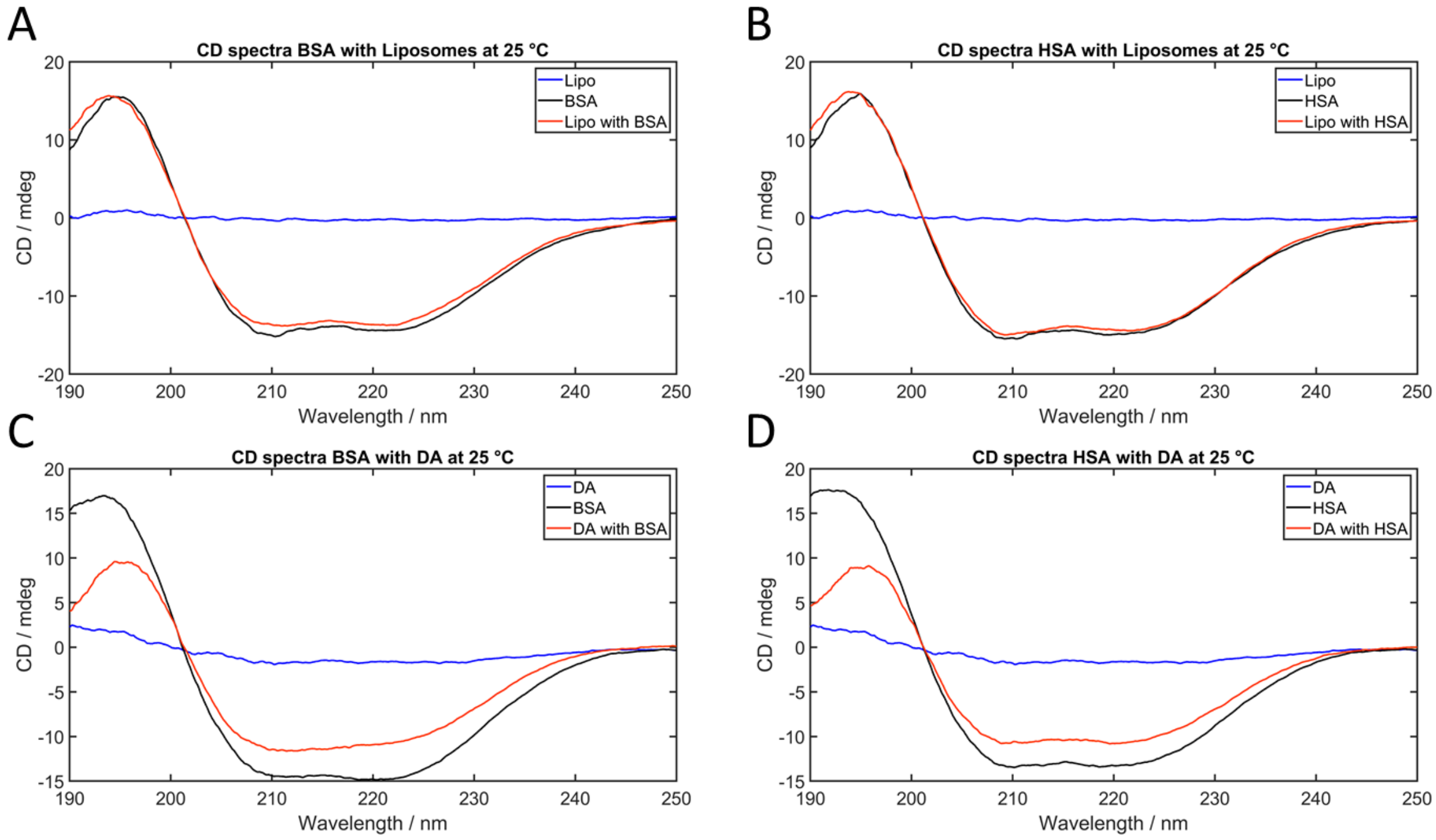

Figure S 18: Far-UV CD spectra of XSA (10 µM) with DPPC/POPS liposomes (1 mM) and DA (50 mM) at 25 °C.

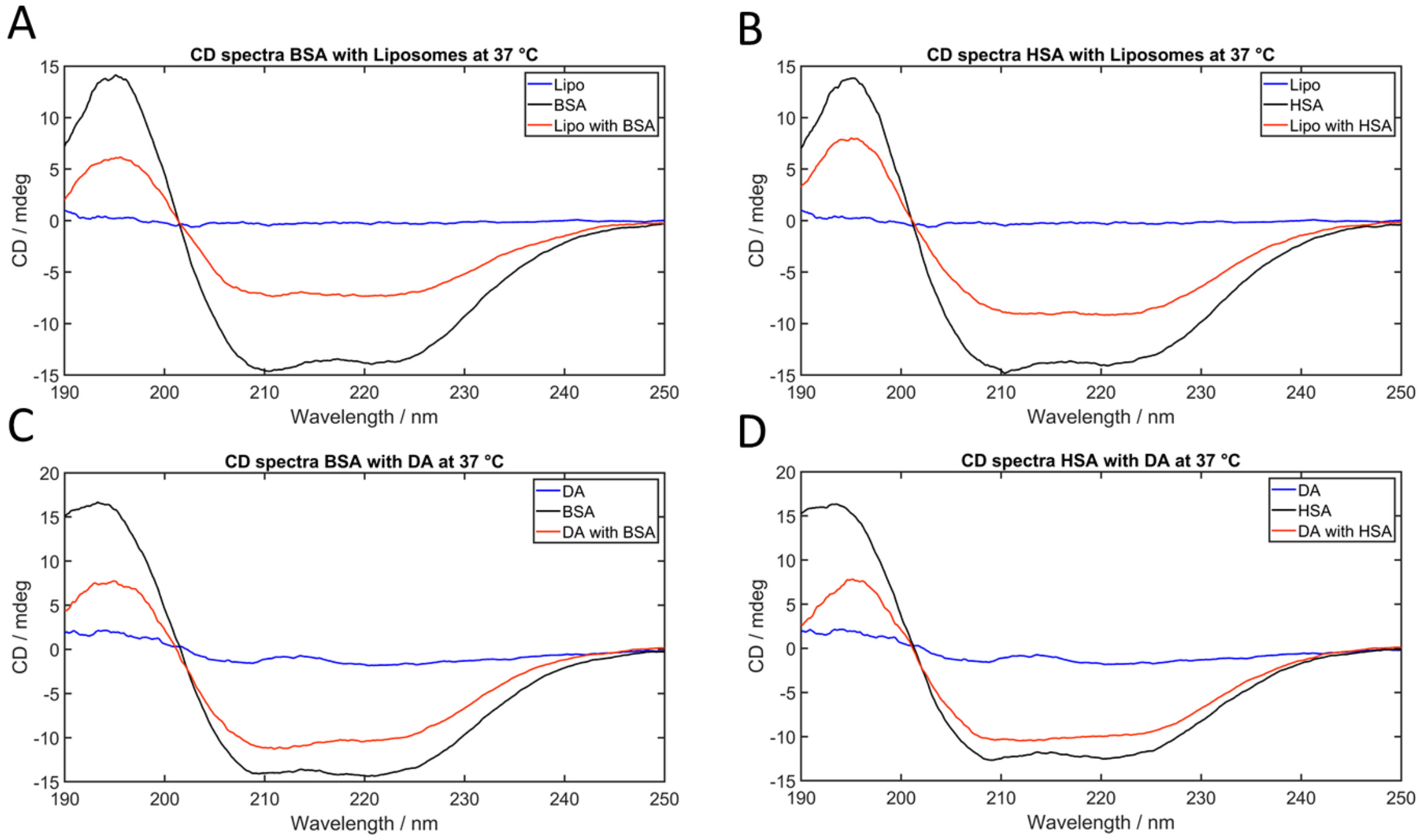

Figure S 19: Far-UV CD spectra of XSA (10 µM) with DPPC/POPS liposomes (1 mM) and DA (50 mM) at 37 °C.

Table S 7: Estimated proportions of secondary structural elements of XSA (in %) in the samples analyzed with CD spectroscopy. The proportions were estimated by spectral fitting in the software BeStSel.[4]

Sample	α-helix / %	β-sheet / %	Turn / %	Other / %	RMSD
BSA 25 °C pure DPBS	55	10.5	7	27.5	0.4305
BSA 25 °C pure water	45.9	14.7	8.6	30.8	0.1974
BSA 37 °C pure DPBS	51.9	12.5	7.1	28.5	0.4439
BSA 37 °C pure water	47.2	12.6	9.6	30.6	0.2065
HSA 25 °C pure DPBS	56.7	10.9	7.4	25.1	0.452
HSA 25 °C pure water	47.4	11.0	10.1	31.4	0.1229
HSA 37 °C pure DPBS	49.3	13.7	6.8	30.1	0.4598
HSA 37 °C pure water	44.8	9.1	10.8	35.2	0.1312
Lipo-BSA 25 °C	46.0	11.7	7.6	34.7	0.2837
Lipo-BSA 37 °C	24.8	19	13.2	43	0.1706
Lipo-HSA 25 °C	52.9	10.7	8.4	28	0.3337
Lipo-HSA 37 °C	28.1	21.8	10.6	39.5	0.2104
DA-BSA 25 °C	41.8	16.6	7.1	34.5	0.2869
DA-BSA 37 °C	40.6	17.7	9.4	32.4	0.2239
DA-HSA 25 °C	37.7	15.8	8.5	38.1	0.2348
DA-HSA 37 °C	37.1	18.6	8.9	35.4	0.2726

Comment on the results of the far-UV CD spectroscopy

The far-UV CD spectra of BSA and HSA exhibit the characteristic minimum with a double peak at 209 and 222 nm which originates from α-helices. Although a peak broadening and slight differences in the peak intensities are visible compared to the ideal α-helix spectrum, the general shape of the spectra are highly consistent with former CD measurements on albumins from other groups.[6-8] Pure XSA shows α-helix proportions of 45-57% depending on the medium and the temperature. These values are slightly lower than those reported in the literature, which might originate from different incubation and measurement conditions, different buffer systems and stabilizers or different fitting protocols. The proportion of α-helices differs slightly between DPBS buffer and water with a generally higher percentage occurring in buffer. Furthermore, HSA exhibits higher α-helix percentages at 25 °C, but BSA has higher values at 37 °C. DA and liposomes both show a small positive CD signal at short wavelengths, the respective spectra were subtracted from the spectra of the mixtures. Buffer and water are mainly not adsorbing but were also subtracted in all sample spectra as background.

With 50 mM DA, α-helix percentage gets slightly reduced for BSA and HSA at 25 and 37 °C and β-sheet content increases slightly, but without much differences between the temperatures. With 1 mM liposomes, α-helix percentage gets slightly reduced at 25 °C first, but it becomes drastically reduced at 37 °C with BSA and HSA (down to 24.8% by 27.1% and down to 28.1% by 21.2%, respectively), while contributions from β-sheets, turns and other structural elements increase at the same time.

At 37 °C the albumin activity for interactions with liposomes might be higher or the approach of the lipid phase transition temperature of the liposomes promote the albumin-liposome interactions and therefore conformational changes of the albumin. The decrease of α-helix and increase of β-sheet content due to the interaction with lipids has been reported very similarly for BSA with cationic lipids.[6]

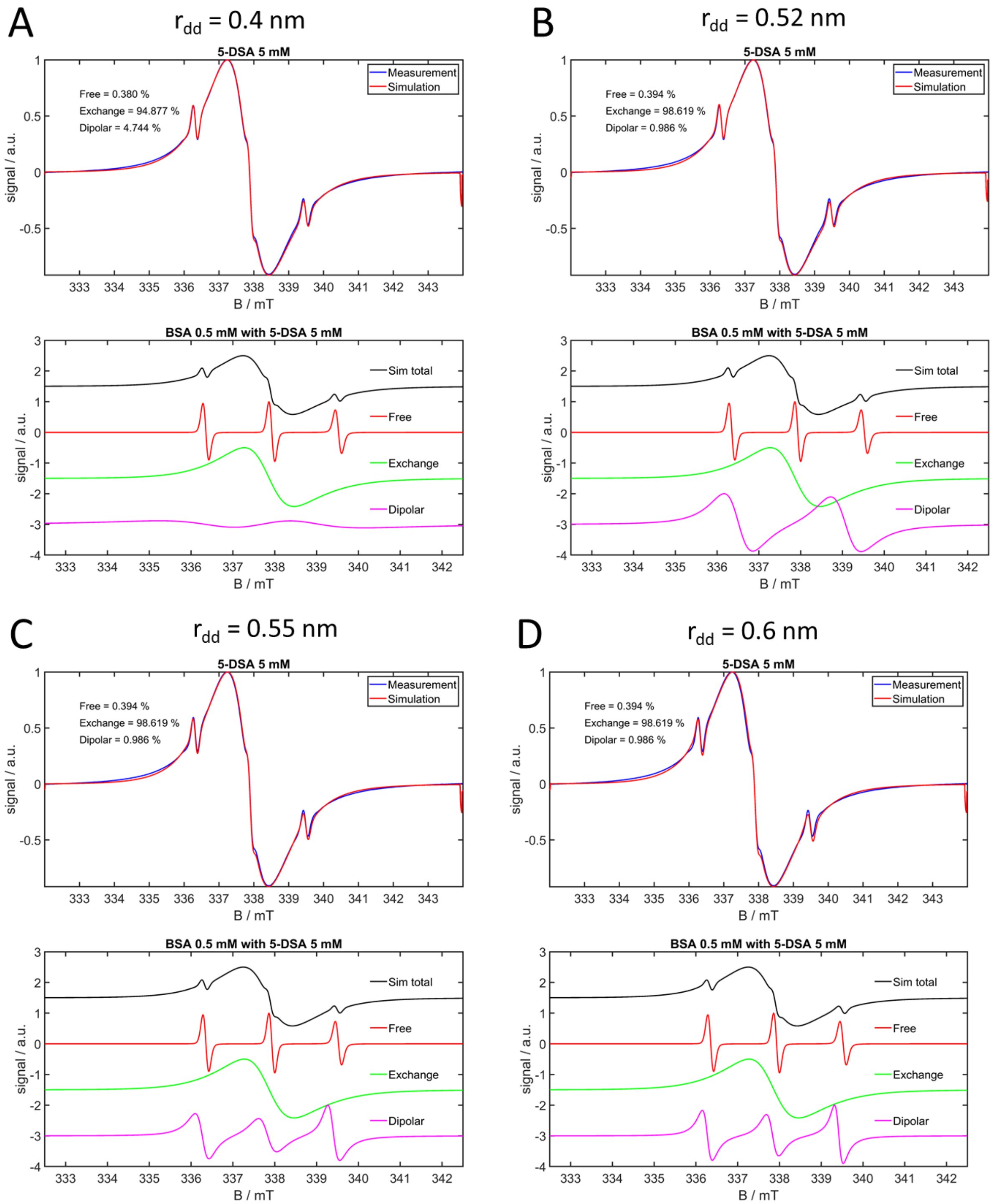

Figure S 20: Simulations of the CW EPR spectrum of 5 mM 5-DSA with exchange (green) and dipolar coupling (purple), applying different spin-spin distances.

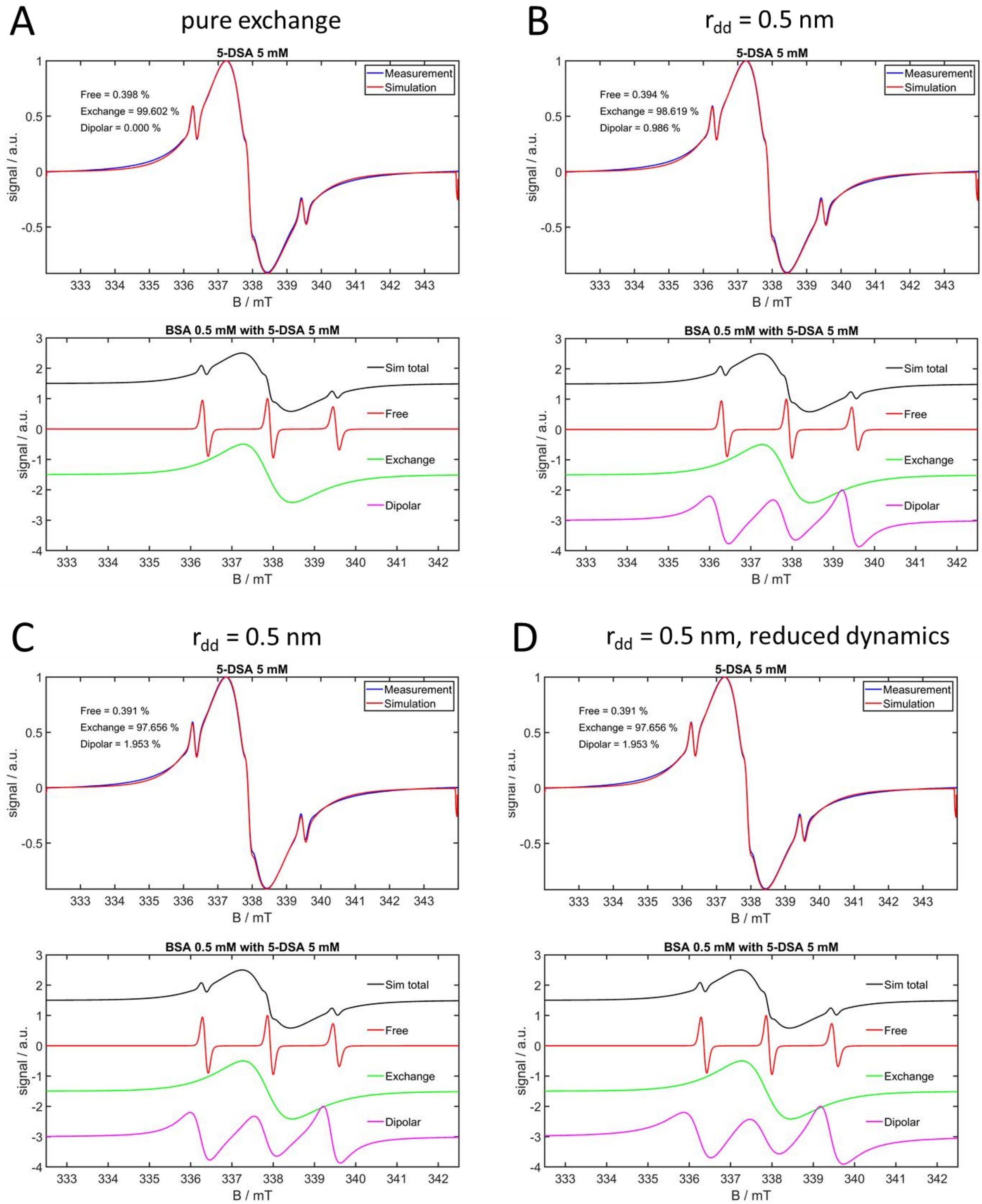

Figure S 21: Simulations of the CW EPR spectrum of 5 mM 5-DSA with pure exchange coupling (A) and mixed exchange (green) and dipolar coupling (purple), using different proportions of the dipole-coupled component (B-C) and reduced rotational dynamics (D).

Table S 8: Influence of dipole-coupled components with different spin-spin-distances and weightings in relation to the exchange-coupled component on the RMSD value of the EPR simulation for 5 mM 5-DSA.

Spin distance / nm	Exchange comp. / %	Dipolar comp. / %	RMSD
-	99.6	0	**1.875 (original)**
0.4	99.1 (1)	0.496	1.879
0.4	98.6	0.986	1.885
0.4	94.9	4.7	1.972
0.45	94.9	4.7	1.972
0.45	98.6	0.986	1.885
0.5	99.1	0.496	1.851
0.5	98.6	0.986	1.85
0.5	97.66	1.95 (τ_c reduced)	**1.827 (best RMSD)**
0.5	94.9	4.7	2.513
0.5	97.66	1.95	1.913
0.5	98.14	1.47	1.871
0.6	98.62	0.986	2.242
0.55	98.6	0.986	1.969
0.52	98.6	0.986	1.914

Comment on the simulation of dynamic exchange coupling and dipolar coupling

The analysis and separation of exchange and dipolar coupling from electron-electron interactions has been investigated by other groups in detail, e.g. by Keller et al..[9]

For the original *Easyspin* simulation of the system 5 mM 5-DSA we used the general function *chili* and the specific function *Sys.Exchange* to simulate dynamic, intermolecular collisional exchange of radicals with a certain exchange frequency, which was 59 MHz in the case of 5-DSA. It was then checked by us, whether dipolar coupling also contributes to the broadening of the spectrum besides exchange broadening. However, to contribute dipolar coupling, the function *Sys.dip* is required which cannot be simulated simultaneously with *Sys.Exchange* in one spin system. While the function *Sys.J* could be used to simulate an isotropic exchange coupling based on the Hamilton operator for electron-electron spin-spin interactions, this function is usually applied on exchange-coupled electron spins of static intramolecular biradicals. It appears to deliver different results than *Sys.Exchange* due to their different simulation nature despite a similar physical origin of SOMO orbital overlap.

In general, *Sys.J* and *Sys.dip* are more frequently used for powder-like EPR spectra in the rigid limit (without any rotational dynamics), requiring the use of the function *pepper*, in which dipoles do not average out as it is usually the case in dynamic regimes. However, we were still able to simulate dipolar coupling with the function *chili* **and** with varying rotational dynamics in *Easyspin*. But due to their incompatibility in one system, we simulated two broadened components via *chili*, the original dynamic, exchange-broadened species and a new dipole-coupled species separately and we let them superimpose

with certain weightings. We did not use *Sys.J* for these simulations and rather focused on *Sys.dip* and *Sys.Exchange* to keep the number of free variable parameters as low as possible. The dipolar component was simulated as a two-spin system with identical *g*, *A* and τ_c tensor values for each electron spin, we let both systems remain dynamic and let each electron spin only interact with its own nitrogen nucleus. We then used an axial dipole tensor to couple the electron spins with each other and firstly calculated the tensor values for different spin-spin distances r_{dd}. Above r_{dd} = 1 nm the spectra were split into a variety of new, sharp peaks or appeared very similar to the free component, but no global broadening occurred and no improvement of the total simulation could be observed. For shorter distances of 0.3 to 0.6 nm, the dipole-coupled component became very broad with a rather simple spectral shape. Hence, we used this distance range instead and optimized the fraction of the dipolar component in relation to the exchange-broadened component.

Indeed, we could reach a slightly lower RMSD value of the total simulation by adding the dipolar coupled component than solely with the exchange-broadened component before, since the outer spectra slopes were slightly affected by the contributing dipolar coupling (see **Figure S 20-21**). By firstly adding 0.99% of dipolar component arising from two spins in the distance 0.5 nm and a resulting axial dipolar coupling constant D_{ax} = 416.32 MHz leading to the dipole tensor [-208.16; -208.16; 416.32], the RMSD value decreased from 1.88 to 1.85. This distance is relatively short but not unrealistic for densely aggregated 5-DSA molecules. Other distances or weightings of the dipolar component lead to an increase of the RMSD. But a small reduction of the radicals' rotational dynamics from τ_c = 0.17 ns to 0.22 ns and a subsequent increase of the dipolar component fraction to 1.95% (leaving 97.66% exchange-broadened and 0.39% free) resulted in an optimum final RMSD value of 1.827. However, we evaluate this final gain of simulation precision as still relatively low while 1.95% is also a very low contribution of dipole-coupled radicals compared to the exchange-coupled ones. Hence, we could validate that the dynamic, intermolecular exchange coupling is clearly the best way to simulate the strong broadening of the spectra arising from the high local 5-DSA concentration, being sufficiently precise in this context. The dipole-coupled contribution was therefore not included in further simulations of this study due to their low percentage and since it is not necessary for the main purpose of the simulations. Finally, the question appears whether the split exchange-coupled and dipole-coupled species make physically sense in this type of aggregated system and whether the simulation in the dynamic regime with *chili* is valid in general. However, answering these questions and more detailed simulation series of dipolar and exchanged-coupled nitroxide systems in a mixture, possibly also with isotropic coupling *J*, would be interesting, but would exceed the scope of this study and were therefore not performed in this context.

Supporting References

(1) Stoll, S.; Schweiger, A. EasySpin, a comprehensive software package for spectral simulation and analysis in EPR. *J Magn Reson* **2006**, *178* (1), 42-55. DOI: 10.1016/j.jmr.2005.08.013 From NLM PubMed-not-MEDLINE.

(2) Freed, J. H. Theory of slow tumbling ESR spectra for nitroxides. In *Spin Labeling: Theory and Applications*, L. J. Berliner, E. Ed.; Vol. 1; Academic Press: New York, 1976; pp 53–132.

(3) Schneider, D. J.; Freed, J. H. Calculating Slow Motional Magnetic Resonance Spectra. In Spin Labeling: Theory and Applications. Berliner, L.J., Reuben, J., Eds.; Springer US: Boston, MA, USA, 1989; pp 1–76.

(4) Micsonai, A.; Wien, F.; Kernya, L.; Lee, Y. H.; Goto, Y.; Refregiers, M.; Kardos, J. Accurate secondary structure prediction and fold recognition for circular dichroism spectroscopy. *Proc Natl Acad Sci U S A* **2015**, 112 (24), E3095-3103. DOI: 10.1073/pnas.1500851112.

(5) Reichenwallner, J.; Michler, S.; Schwieger, C.; Hinderberger, D. Human Serum Albumin Loaded with Fatty Acids Reveals Complex Protein-Ligand Thermodynamics and Boleadora-Type Solution Dynamics Leading to Gelation. *Journal of Physical Chemistry B* **2025**, *129* (14), 3571-3589. DOI: 10.1021/acs.jpcb.4c08717.

(6) Charbonneau, D. M.; Tajmir-Riahi, H. A. Study on the interaction of cationic lipids with bovine serum albumin. *J Phys Chem B* **2010**, *114* (2), 1148-1155. DOI: 10.1021/jp910077h.

(7) Juarez, J.; Taboada, P.; Mosquera, V. Existence of different structural intermediates on the fibrillation pathway of human serum albumin. *Biophys J* **2009**, *96* (6), 2353-2370. DOI: 10.1016/j.bpj.2008.12.3901.

(8) Luong, T. Q.; Verma, P. K.; Mitra, R. K.; Havenith, M. Do Hydration Dynamics Follow the Structural Perturbation during Thermal Denaturation of a Protein: A Terahertz Absorption Study. *Biophysical Journal* **2011**, *101* (4), 925-933. DOI: 10.1016/j.bpj.2011.05.011.

(9) Keller, K.; Ritsch, I.; Hintz, H.; Hülsmann, M.; Qi, M.; Breitgoff, F. D.; Klose, D.; Polyhach, Y.; Yulikov, M.; Godt, A.; et al. Accessing distributions of exchange and dipolar couplings in stiff molecular rulers with Cu(ii) centres. *Phys Chem Chem Phys* **2020**, *22* (38), 21707-21730. DOI: 10.1039/d0cp03105d.
